# Impact analysis of institutional framework and policy preferences with Bayesian structural equation

**DOI:** 10.1371/journal.pone.0358701

**Published:** 2026-09-22

**Authors:** Song Jin, Liguo Chang, NanNan Zhang, Ye Li

**Affiliations:** 1 Chongqing Creation Vocational College, Chongqing, China; 2 Institute of Economics and Management, Kyrgyz State University named after I. Arabaev, Bishkek, Kyrgyz Republic; 3 College of Marxism, East University of Heilongjiang, Heilongjiang, China; 4 School of Business Administration, Guangzhou Institute of Science and Technology, Guangdong, China; 5 College of International Studies, Southwest University, Chongqing, China; 6 College of Marxism, Tibet University, Lhasa, Tibet Autonomous Region, China; City University of Hong Kong, HONG KONG

## Abstract

Mediation analysis is able to deeply analyze the finer mechanism of the path of exposure variables leading to outcome variables and has received extensive attention in many fields in recent years. In order to further analyze China’s energy cooperation with neighboring countries, this paper divides the two stages of policy formulation and implementation. It connects the logical chain of “cooperation policy preferences at the decision-making level, final cooperation policy preferences to energy trade and investment cooperation.” By constructing a partial Bayesian structural equation model, we verify the mediating effect played by final cooperative policy preferences. The mediation analysis based on structural equation modeling provides the possibility to analyze the complex structure among variables, including the mediation effect. And Bayesian SEM can provide a more flexible program for the construction and estimation of complex models. In addition, this paper explores the statistical analysis strategy of mediation analysis of Bayesian structural equation modeling and its statistical performance under different data characteristics through example analysis. The empirical results show that policy preferences play a significant mediating role between institutional frameworks and energy cooperation outcomes. Specifically, the Bayesian SEM estimates a positive indirect effect of 0.305, with a standard error of 0.123, a 95% confidence interval of (0.061, 0.552), and a significance level of p = 0.006. This indicates that institutional arrangements promote energy cooperation mainly by shaping policy preferences rather than through direct effects alone. Compared with classical regression-based mediation, classical SEM, Bayesian regression, and other alternative approaches, Bayesian SEM demonstrates stronger performance in handling latent-variable uncertainty, interval estimation, and parameter stability. Across the 12 estimation methods employed in this study, the estimated indirect effects range from 0.271 to 0.312 and remain consistently significant, confirming the robustness of the mediation mechanism. This indicates that while maintaining directional consistency, it offers more robust and reliable handling of latent variables and uncertainty.

## 1. Introduction

As a large oil and gas importing country, China has four major oil and gas import corridors in its neighborhood that are of great significance to its energy import security. Cooperation with the neighboring countries is necessary to ensure the security of China’s energy imports and supply [1]. In recent years, China’s new energy exports in the international market have occupied an important position [2]. Furthermore, as the largest provider of renewable energy equipment globally, China can assist its neighbors in realizing the enormous potential of renewable energy [3,4] and in exchanging pertinent expertise regarding the development, execution, and modification of policy goals.

As a kind of national behavior, international energy cooperation is affected by the foreign policy preferences of each country. From the perspective of the cooperation system, energy cooperation between China and neighboring countries is not only influenced by China’s side but also by the foreign policy preferences of neighboring countries [5]. From the above, it is clear that there are multiple layers of complex relationships in international energy cooperation relationships. In some related studies, it is often necessary to establish the causal connection among the stimulus and the resulting event in order to predict the study outcome from the factors or combinations of the factors for practical applications like influencing social decision-making [6]. When both dependent and independent variables are specified in a study’s structure, the independent variable may use a third variable, known as the mediating variable, to indirectly influence the variable that is dependent [7]. The analysis of mediating variables is an important part of a comprehensive and in-depth investigation of causal mechanisms or pathways of influence [8]. Using suitable statistical techniques, mediation analysis examines whether the mediating variable and the amount of the explained effect can account for the independent variable’s predictive impact on the dependent variable [9].

To determine the direct and indirect paths of the independent variable’s effect on the dependent variable, as well as to assess the link between the explicit, latent, or error parameters contained in the model, the SEM combines factor analysis and path analysis [10]. SEM can use global modeling to illustrate the link between variables. Second, a variety of causal models can be identified, estimated, and validated using SEM. Unlike traditional statistical techniques, SEM allows for the simultaneous handling of multiple variables that are dependent. Second, the model’s fit is another factor that must be carefully taken into account when studying complex multilevel data. With the advancement of mathematical and statistical methods and computer technology, Monte Carlo Markov Chain (MCMC for short) sampling and simulation techniques and statistical methods under the Bayesian framework [11] are becoming more and more commonly used in various fields. The main attraction of the Bayesian approach is its formalized use of prior information to make full use of external information and improve estimation performance [12].

Despite the growing body of literature on international energy cooperation, several critical gaps remain. First, most existing studies focus primarily on observable economic indicators or institutional arrangements, while the role of latent policy preferences—such as energy security concerns, environmental priorities, and technological complementarity—is often treated as exogenous or insufficiently modeled. This limits the ability to uncover the underlying transmission mechanisms through which institutions influence cooperation outcomes. Second, mediation effects in this field are typically examined using regression-based approaches or classical structural equation modeling, which are sensitive to measurement error, model misspecification, and small sample sizes. These limitations are particularly problematic in cross-national policy research, where data are heterogeneous and latent constructs are difficult to measure directly. Third, although Bayesian structural equation modeling provides a flexible framework for handling latent variables and parameter uncertainty, its application to mediation analysis in international energy cooperation remains scarce. In particular, there is a lack of empirical studies that integrate text-based policy measurement, latent variable modeling, and Bayesian inference within a unified analytical framework.

To address these gaps, this study develops a Bayesian SEM–based mediation framework that explicitly models policy preferences as latent mediators, incorporates uncertainty through Bayesian estimation, and operationalizes policy dimensions using text-based indicators. By doing so, the paper provides a more robust and transparent approach to analyzing the mechanism through which institutional frameworks influence energy cooperation outcomes. This study adopts the country–year as the primary unit of analysis, where each observation represents a national policy context in a given year. Within this framework, institutional arrangements are conceptualized as formal policy structures, while policy preferences are treated as latent constructs reflecting strategic policy orientations derived from official documents. Drawing on political economy and policy process theories, the model assumes that institutional frameworks shape cooperation outcomes indirectly through their influence on policy priorities. This conceptualization provides a theoretically grounded basis for the mediation structure tested in this study. The hypotheses of this study are stated as follows.

H1: The policy and regulatory framework has a significant effect on policy preferences. H2: Policy preferences have a significant effect on energy cooperation outcomes.

H3: Policy preferences mediate the relationship between institutional framework and energy cooperation.

In conclusion, it seemed obvious to use Bayesian SEM to build mediation analysis. Based on global modeling of SEM, Bayesian SEM has naturally emerged as a key methodological source in mediation effect analysis. It uses MCMC sampling techniques to increase computational speed and, when feasible, incorporates external prior information. In this research, we evaluate the outcome link between China and neighboring countries’ energy cooperation using Bayesian structural equation modeling. We set the institutional framework as the independent variable, policy favoritism as the mediating variable, and energy cooperation as the dependent variable. After collecting and pre-processing the data, multiple dimensions under one variable were quantified. Finally, the established data were validated and analyzed using several different models. Comparison reveals that Bayesian SEM has higher confidence interval coverage and more robust results with small sample data. The SEM approach leads to smaller standard errors and improved parameter estimation accuracy. The main innovations of this paper are the following three points.

(1) A Bayesian SEM mediation method integrating traditional mediation analysis with counterfactual causality frameworks has been designed. This method employs MCMC to handle complex models with multiple latent variables, simplifying computations for factor structure data while incorporating prior information to enhance parameter estimation accuracy.
(2) A multidimensional quantitative indicator system was constructed based on energy and integration factors, employing TF-IDF to assess keyword significance.
(3) Exploratory factor analysis was used to evaluate the validity and reliability of the indicators. Combined with confirmatory factor analysis and structural equation modeling, a comprehensive analytical pathway was established to quantify the impact of policy preferences on energy cooperation mechanisms.

Through multi-model comparisons, the system contrasts the differences between Bayesian SEM mediation and regression mediation with classical SEM, validating its advantages under complex structures and small sample conditions.

## 2. Related work

### 2.1. Institutions and roles in energy cooperation

The impact of renewable energy, including wind, biodiesel, and solar PV, on local sustainable development is significant, with positive effects on employment, income generation, diversification of the local economy, and social cohesion. Rio et al. [13] use a combination of theoretical analysis and field case studies, combining quantitative and qualitative methods, including interviews with local stakeholders and statistical analysis of economic and social indicators. This resulted in a comprehensive and systematic assessment of the economic and social benefits of renewable energy projects at the local level rather than the national level. A new framework for analyzing the socio-physical interactions of regional renewable energy deployment compares the policy environment, governance mechanisms, and infrastructure needs for renewable energy deployment in different regions of Italy and the UK [14]. This illustrates the importance of regional institutions, governance capacity, and infrastructure.

China likewise faces numerous obstacles and challenges in fostering global collaboration in the renewable energy sector. Three phases of energy policy development can be identified from the analysis of China’s energy policy from 1981 to 2020. China concentrated on increasing energy efficiency between 1981 and 2000 [15]. It focuses on growing energy security challenges between 2001 and 2010. Then, between 2011 and 2020, China began the transition to a low-carbon economy by integrating climate change into energy policy. Zhao et al. [16] offer a thorough evaluation of China’s involvement in global energy cooperation as it stands today, together with suggestions for future policy from the standpoint of critical analysis. Regional cooperation, cooperation among developing nations, and collaboration between developing and developed nations are the three mechanism types they put out. Christopher M et al. [17] provide an in-depth analysis of the policy motivations, industrial layout, and business models for renewable energy development in China. They use a dichotomous political economy research methodology to analyze information from multiple sources covering a two-year period. They illustrate three main motivations for China’s renewable energy push. These are energy security, environmental well-being, and strategic industrial development. Wei et al. [18] use a political economy perspective to provide a new policy paradigm perspective and analytical framework for the political economy context of China’s low-carbon energy transition, especially in the wind and solar industries. They offer a new policy paradigm for China’s low-carbon energy transition in addition to going beyond the conventional “state-led” theory to show how internal power struggles and external shocks affect policy paradigm shifts. Additionally, they suggest replacing the existing development model, which was focused only on capacity increase, with a new policy paradigm. Focusing on the case of China, Duan et al. [19] investigated the correlation between energy and water resources in international energy trade. A mixed unit IOA analysis was used to reveal the energy-water resource parallelism. ENA was used to reveal the resource pathways in global trade. Qi et al. [20] used the China Global Energy Model (C-GEM) for scenario analysis and quantitative prediction to analyze the policy effects by comparing the differences in emissions and energy consumption between different policy scenarios. It reveals that emission reductions in the energy sector may lead to increased emissions in other non-energy sectors as a result of the “leakage” effect, and emphasizes the importance of comprehensive cross-sectoral emission reduction policies. In order to analyze the current situation of China’s promotion of international cooperation on energy transition, a strategic positioning of international cooperation and a cooperation framework based on China’s perspective are proposed [21]. Using SWOT strategic analysis, China’s multiple roles in international cooperation on energy transition are proposed, and four major institutional frameworks for international cooperation are designed. Zhang et al. [22] innovatively combined the analytical framework of institutional economics to specifically analyze how the governance model affects the implementation effect of market mechanisms. Moreover, they clearly put forward the view that it is difficult for market reform to achieve the expected efficiency and emission reduction effect under the current institutional background.

### 2.2. Mediation analysis

In mediation analysis, the methodology has progressively shifted from defining and reporting indirect effects to robust estimation under complex measurement and multi-path scenarios. Early studies clarified the concept of mediators, distinguished them from moderators and confounders, and provided standard equations and reporting guidelines for single-mediation models [23]. They emphasized the value of randomized experiments and bootstrap intervals for inference, establishing a common language and evaluation framework for subsequent empirical research. Building on this foundation, scholars further extended the framework to multiple mediators [24]. To distinguish between total indirect effects and specific indirect effects, statistical procedures were developed to compare multiple mediation pathways within the same model. Distributional products and bootstrap methods were employed to enhance significance testing and interval coverage, thereby improving practical applicability and testing power. Simultaneously, to address measurement errors in latent variables and the demands of complex structural models, PLS-SEM provides a systematic operational paradigm from measurement to structure [25]. This encompasses critical stages such as reflective and formative measurement, structural path evaluation, and mediation and moderation analysis, enabling the assessment and validation of mediating mechanisms under data conditions closer to real-world applications.

Muthén [26] extended traditional SEM into a broader family of statistical modeling frameworks by adopting latent variables as a unified language. This approach encompasses random effects, variance components in hierarchical data, finite mixtures and latent classes, and discrete-time survival analysis. Its application emphasizes the integration of statistics and psychometrics. This methodology transcends the traditional limitations of SEM, which was previously confined to measuring error and multi-indicator latent constructs. Preacher et al. [27] identified two limitations in traditional multilevel linear models (MLM) for mediation analysis. One involves confounding between-group and within-group effects, leading to biased indirect effects. The other is the difficulty in modeling upward pathways. Therefore, they proposed using multilevel structural equation modeling (MSEM) to unify and extend existing multilevel mediation models. Within a two-level framework, MSEM decomposes Level-1 variables into latent components representing between-group and within-group effects, estimating corresponding paths separately to yield unconfounded indirect effects. MSEM also integrates Level-2 outcome variables and upward effects into a unified estimation. Krull et al. [28] combined mediation analysis with multilevel models to address unit selection and standard error bias in clustered data, proposing multilevel mediation frameworks tailored for designs such as 1-1-1, 2-1-1, and 2-2-1. This method replaces single-level OLS with multilevel models estimated via EB/ML, estimating paths at different levels and calculating indirect effects accordingly. Simulations reveal that the standard errors of indirect effects in single-layer approaches are often biased downward by over 20%, with this bias becoming more pronounced under high within-group correlations and large group sizes. To enable effective inference of structural parameters in the presence of complex disturbance parameters, a universal paradigm combining orthogonal scoring and cross-fitting is designed to counteract first-order bias arising from regularization bias and overfitting [29]. It constructs Neyman-orthogonal moments to reduce sensitivity to disturbance estimation errors and employs cross-fitted data splitting. The estimator is proven to achieve root-N convergence and approximate normality under weak conditions, enabling it to estimate disturbance functions in conjunction with various ML learners. Building upon the product coefficient method, Preacher et al. [24] derived the asymptotic variance for the multivariate Delta method and advocated using the autocovariance method and distribution-product method to construct intervals and improve small-sample coverage. They also noted that allowing residual correlation among mediators is advisable in multiple mediation contexts. Their two-step strategy involves first testing the total indirect effect, followed by testing specific effects. Simulation and empirical results demonstrate that the distribution-product and bootstrap methods exhibit higher power and better control of Type I error rates compared to traditional causal steps or Sobel tests.

Overall, existing mediation analysis methods have evolved from simple regression-based approaches to more complex multilevel and SEM-based frameworks. However, these approaches still face several limitations. Regression-based mediation is sensitive to measurement error and cannot adequately capture latent constructs, while classical SEM methods rely heavily on large-sample assumptions and may produce biased estimates under small sample conditions. Although recent advances incorporate multilevel structures and bootstrap techniques, the treatment of parameter uncertainty remains limited. These shortcomings highlight the need for a more flexible framework that can simultaneously address latent variable measurement, complex mediation structures, and uncertainty—motivating the adoption of Bayesian structural equation modeling in this study.

### 2.3. Structural equation model

SEM is a multivariate statistical method that accounts for measurement error and is used to estimate and verify the network of causal path links between latent and observable variables [30]. Path analysis, confirmatory factor analysis, latent growth curve modeling, and comprehensive SEM modeling are examples of SEM sub-models. Piotr et al. [24] systematically reviewed the history of SEM, especially the origins of factor analysis and path analysis. They provide an in-depth analysis of the statistical and philosophical controversies of SEM, including the issues of null hypothesis testing, model omission of variables, multicollinearity, and model fit evaluation, and emphasize the importance of theoretical assumptions for model construction.

Tenko et al. [31] then address important issues in applied SEM research, such as sample size, parameter estimation methods, model fit assessment, and stochastic capitalization. They used the asymptotic distribution-free (ADF) estimation method and a new strategy for model fit assessment (non-centrality, confidence interval method) to illustrate that the method relies on large sample data and performs poorly in small sample conditions. For the treatment of large-scale survey data, Bengt O et al. [32] also explores the implementation of SEM methods under complex sample design data. Two methods are proposed to handle complex sampling data. The first is aggregation analysis, which adjusts the standard error and goodness of fit. The second is decomposition analysis, which increases the parameters of the complex sampling structure. Monte Carlo simulations confirm that these methods improve the accuracy and statistical validity of SEM for complex sample design data.

In addition, SEM has been extended to the field of family lineage data analysis, enabling the simultaneous analysis of multiple genetic variants in relation to multiple related phenotypes [33]. The problem is that the implementation of the model is highly dependent on software tools. For latent variable interaction estimation methods, especially the Latent Moderated Structural Equations (LMS) method. Julie et al. [34] provide methods for estimating latent variable interactions that can effectively handle measurement errors and improve statistical efficacy. At the same time, they extend to methods such as nonlinear relationships, quadratic effects, and growth factor interaction effect estimation. This can effectively avoid the insensitivity of traditional methods to measurement error and is suitable for dealing with interactions between latent variables. Bollen et al. [35] also emphasize the advantages of latent variable models in solving problems of measurement error and multiple equation modeling, especially their application in behavioral sciences when quantifying complex relationships of latent concepts that are difficult to observe directly, such as pain perception and sleep quality.

A dynamic structural equation modeling (DSEM) framework for analyzing dense longitudinal data such as daily questionnaires and ecological transient assessment data, that captures dynamic relationships [36] between observed and latent variables over time. To facilitate the integrated study between individual differences and temporal dynamics, it integrates time series analysis, multilevel modeling, SEM, and time-varying effects modeling [37]. Mueller et al. [38] compiled best practices for performing SEM investigations, such as initial model conception, parameter identification and estimate, model fit assessment, and potential model adjustments, in order to enhance the caliber and dependability of SEM studies. This provides a detailed SEM analysis process and practical advice. When it comes to causal inference, SEM faces some controversies and problems. In response to this, Bullock et al. [39] emphasize that SEM does not confirm causality through statistical methods alone but requires carefully designed experiments and the fulfillment of specific conditions in order to make preliminary causal inferences. If it is not used carefully, it is easy for researchers to incorrectly interpret correlations as causal relationships.

In summary, while SEM provides a powerful framework for modeling complex relationships among latent variables, its classical estimation methods exhibit several constraints, including sensitivity to sample size, distributional assumptions, and model specification. Extensions such as LMS and DSEM improve flexibility but often increase computational complexity and remain dependent on large datasets. These limitations suggest that, although SEM is suitable for mediation analysis, its effectiveness can be further enhanced by incorporating Bayesian inference to improve robustness and parameter estimation under uncertainty.

### 2.4. Mediating effects and structural equation modeling in energy policy

In the context of the global response to climate change and the promotion of energy transition, the mediating role of energy consumption and its related variables in environmental governance has gradually been paid attention to [40,41]. Taner [42] used Bootstrap Explicit Variable Structural Equation Modeling (SEM) to explore how financial development affects carbon emissions through the mediating variable of energy consumption. And study how to integrate the financial system into the national energy modeling system. Taking the data of the United States from 1990 to 2021 as a case study, they not only focus on the direct effect but also emphasize the indirect effect of finance on carbon emissions. At the urban micro level, the mediating effect of energy is manifested as changes in the natural environment. Wang et al. [43] used satellite imagery to obtain land cover and surface temperature (LST) for the period of 1990–2018 in Nanjing and investigated how land cover changes lead to an increase in building electricity consumption through surface temperature and thus with a focus on seasonal differences. Mediated effects analysis was used to test the mediating role of LST between LUCC and electricity consumption [44].

Energy possesses a similar mediating path between business management and consumer behavior. Mehrab et al. [45] explored the factors influencing consumers’ willingness to use renewable energy in Pakistan, focusing on the mediating role of attitudes between perceived value and final purchase intentions. They based their conceptual framework on the Theory of Planned Behavior (TPB) and the Integrated Technology Acceptance Model (UTAUT). And 497 sample data were collected through a structured questionnaire. Finally, Structural Equation Modeling (SEM) was used to analyze the relationship between variables such as social media exposure, perceived ease of use, awareness, and cost. Muhsin et al. [46] used Partial Least Squares Structural Equation Modeling (PLS-SEM) based on data from 189 Turkish industrial firms to study how energy management system (EMS) affects firm performance through environmentally friendly energy consumption (PEC) and its performance (PECP) and to analyze the moderating role of environmental awareness (EA). It is found that EMS contributes more to PECP when the moderating variable, environmental awareness, is higher.

In international cooperation, institutional and technological innovations are recognized as intermediary guarantees for achieving energy efficiency. Green public procurement (GPP) was first identified as a key intermediary between BRI and renewable energy development in a study by Xu [47]. They investigated the contribution of the Belt and Road Initiative (BRI) to the growth of renewable energy in the countries along the route, and in particular the intermediary mechanism of Green Public Procurement (GPP) in it. The pathways by which policy initiatives are translated into substantial environmental benefits through administrative instruments are emphasized. Meanwhile, Wu et al. [48] analyzed the spatial and temporal evolution of carbon emission efficiency (CEE) in 60 Belt and Road countries to explore the mechanism of renewable energy investment and resource endowment on CEE. The super-efficient SBM model is used to assess the static level of CEE, and the Global Malmquist-Luenberger (GML) index is used to measure the dynamic evolution. Spatial Durbin Models (SDM) and Mediated Effects Models were then used to test spillover effects and driving mechanisms. They considered spatial correlations and found positive spillovers from renewable energy investments and a negative effect of resource endowments.

### 2.5. Bayesian structural equation modeling in social sciences

In recent years, Bayesian structural equation modeling (Bayesian SEM) has gained increasing attention in social science research as an extension of classical SEM frameworks [49]. By integrating Bayesian inference with latent variable modeling, Bayesian SEM allows researchers to incorporate prior information, explicitly model parameter uncertainty, and obtain full posterior distributions of model parameters rather than point estimates alone.

Empirical studies in psychology [50], education [51], and sociology [49] have demonstrated that Bayesian SEM is particularly advantageous in situations involving small or moderate sample sizes, complex hierarchical structures, or latent constructs measured with error. Compared with frequentist SEM [52], Bayesian SEM has been shown to provide more stable parameter estimates, improved interval coverage, and greater robustness to model misspecification. These properties make it well suited for policy and institutional research, where theoretical constructs are often multidimensional and data availability is limited.

Despite these methodological advantages, the application of Bayesian SEM in international energy cooperation research remains limited. Most existing studies continue to rely on regression-based or frequentist SEM approaches, with insufficient attention to latent policy mechanisms and uncertainty propagation. Therefore, there is a clear need to integrate Bayesian SEM into this field to provide more reliable mediation estimates and a deeper understanding of policy transmission mechanisms. While macro-level theories such as political economy and environmental governance emphasize the role of institutional priorities and strategic preferences, their empirical operationalization remains challenging. This study bridges this gap by linking these abstract constructs to text-based indicators derived from policy documents. Although such measures cannot fully capture the complexity of policy preferences, they provide a systematic and replicable approximation of institutional emphasis, enabling the integration of qualitative policy content into quantitative modeling frameworks.

## 3. Method

**Fig 1:**
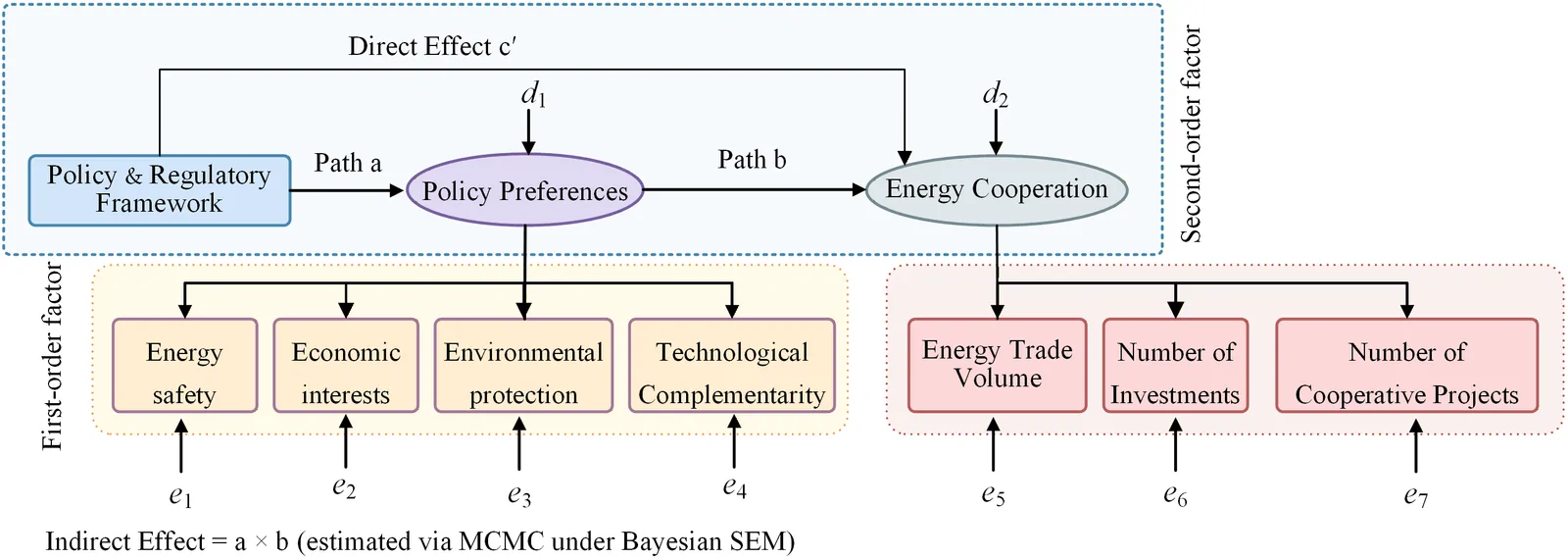
The structure of Bayesian Structural Equation Modeling.

Although the classical regression method is applicable to mediation analysis in a wide range of situations, it exhibits certain limitations as models and data types become more complex. For the Bayesian structural equation model mediation analysis method, it can handle complex data types containing latent and observed variables, multiple latent variables, and latent variables. The MCMC iterative technique combined with the Bayesian method makes the computational process of data containing factor structures simpler. Second, Bayesian structural equation modeling mediation analysis can incorporate a priori information on expertise in some cases, making the estimation results more accurate. The Bayesian SEM structural diagram for this paper is shown in Fig 1.

We assume that the policy and regulatory framework can influence energy cooperation through policy preferences. Therefore, as shown in Fig 1, the policy and regulatory framework, policy preferences, and energy cooperation serve as the independent variable, mediating variable, and dependent variable, respectively. All are second-order factors composed of different first-order factors. The first-order factors are latent variables directly reflected by a set of observed indicators, such as questionnaire items. For example, energy security, economic interests, environmental protection, and technological complementarity in the figure serve as first-order factors for policy preferences.

### 3.1. Bayesian structural equation model

In this study, prior distributions were specified following a weakly informative Bayesian strategy, which is commonly adopted in applied social science research when strong prior knowledge is unavailable. Regression coefficients and intercepts in both the measurement and structural components were assigned normal priors with mean zero and relatively large variance, allowing sufficient flexibility while preventing extreme parameter values. Variance and covariance parameters were assigned inverse-gamma or inverse-Wishart priors to ensure positive definiteness and computational stability. Following standard practice in Bayesian SEM, regression coefficients are assigned weakly informative normal priors, which provide regularization while avoiding overly restrictive assumptions. Variance parameters are assigned inverse-gamma priors to ensure positivity and computational tractability. This specification is consistent with commonly adopted Bayesian SEM frameworks.

These priors serve primarily as regularization devices rather than sources of substantive information, enabling stable estimation under limited sample sizes and complex latent-variable structures. To assess robustness, alternative prior hyperparameter settings were tested, and the resulting posterior estimates of key mediation effects remained qualitatively consistent. This indicates that the main conclusions are not driven by specific prior choices.

The prior distributions of the model parameters and techniques for MCMC-based posterior distribution summarization that combine prior knowledge and likelihood functions are the primary elements of Bayesian structural equation model estimation. In Bayesian statistics, a parameter is given the prior distribution to reflect the prior knowledge that has been updated with the observed data. This results in a posterior distribution of the possible values of that parameter, which can be summed up using point and interval estimation summaries. Equation 1 initially illustrates the measurement model in the continuation of the research of Bayesian estimation for SEM based on Bayesian theory.


$$\begin{array}{c} y=\alpha +\wedge \eta +K\text{x}+\in \end{array}$$ (1)

where y stands for the vector of observable random vectors. α is a vector of measurement intercept terms, and ∧ is the factor loading matrix. Eta represents the vector of latent variables. k represents the regression coefficient matrix relating the observable variable x and the latent variable y. The monotonic vector with the covariance matrix Ξ is denoted by ε. Equation 2 represents the structural model.


$$\begin{array}{c} \eta =\nu +\text{B}\eta +\Gamma \text{x}+\zeta \end{array}$$ (2)

where the structural intercept terms’ vector is denoted by ν. B and Γ are structural coefficient matrices. The vector of covariance matrices Ψ is denoted by ζ.

Bayesian analysis uses either informative or uninformative priors. We adopt conjugate priors in which the intercept and regression coefficients have normal priors and the residual variance has an inverse-gamma prior. The structural parameter vector containing the unknown elements of α,ν,  ∧, B,Γ,K that follow a normal distribution is θnorm={α,ν,  ∧, B,Γ,K}. Thus, θIW={Ξ,Ψ} indicates that the error term has an inverse gamma distribution, as shown by Equation 3.


$$\begin{array}{c} \theta norm\sim N(\mu ,\Omega ) \end{array}$$ (3)

The mean and variance hyperparameters of the normal prior are expressed by the symbols μ and Ω, respectively. Equation 4 illustrates the variance and covariance of Ξ and Ψ, assuming a previous distribution of IW2.


$$\begin{array}{c} \theta IW\sim IW(R,\delta ) \end{array}$$ (4)

If δ>q−1, where q is the number of variables observed, and R is a positive definite matrix. The inverse gamma distribution’s informativeness will vary depending on the values of R and δ that are specified.

After setting the prior, update it with data to obtain the posterior. Conjugate priors yield related posteriors, while non-conjugate cases require MCMC computation. Let π(θ) be the prior distribution ofθ, and let p(x|θ) represent the probability function of the sample. Equation 5 displays the posterior distribution of θ.


$$\begin{array}{c} \pi \left(\theta |x\right)=\frac{p\left(x|\theta \right)\pi (\theta )}{\int p\left(x|\theta \right)\pi (\theta )d\theta } \end{array}$$ (5)

This method centers on computing the posterior expectation of the function h(θ), as shown in Equation 6.


$$\begin{array}{c} E\left[h(\theta )|x\right]=\int h(\theta )\pi \left(\theta |x\right)d\theta =\frac{\int h(\theta )p\left(x|\theta \right)\pi (\theta )d\theta }{\int p\left(x|\theta \right)\pi (\theta )d\theta } \end{array}$$ (6)

If h(θ)=θ, the above equation is the posterior expectation of θ. To estimate population means, variances, and quantiles, we perform Gibbs sampling on the joint posterior distribution. After obtaining sufficient samples, we use the sample mean, sample variance, and sample quantiles as the corresponding Bayesian estimates. To ensure model identifiability, partially unidentified parameters are often fixed. Within the Bayesian framework, these quantities can be treated as latent/missing data and incorporated into the observed dataset y through data augmentation. Many samples are drawn from [θn,θIW, η|y] by the following Gibbs sampling with current values $\eta^{\left(s\right)}$, $\theta_{norm}^{\left(s\right)}$, and $\theta_{IW}^{\left(s\right)}$ in the (s+1)-th iteration.

Algorithm 1 demonstrates the algorithmic pseudocode for estimating Bayesian SEM. Gibbs sampling is employed to update parameters within the model, including latent variables and regression coefficients. At each step in the s iterations of Gibbs sampling, each component of $\eta^{\left(s\right)}$, $\theta_{norm}^{\left(s\right)}$, and $\theta_{IW}^{\left(s\right)}$ is updated one by one, given the most recent values of the other components. In the (s+1) th iteration of Gibbs sampling, $\eta^{\left(s+\text{1}\right)}$ is first updated from the conditional distribution based on the current $\theta_{norm}^{\left(s\right)}$ and $\theta_{IW}^{\left(s\right)}$. Then the $\theta_{norm}^{\left(s+\text{1}\right)}$ is updated conditional on the updated $\eta^{\left(s+\text{1}\right)}$ and the current $\theta_{IW}^{\left(s\right)}$. Finally, the $\theta_{IW}^{\left(s+\text{1}\right)}$ is updated conditional on the updated $\eta^{\left(s+\text{1}\right)}$ and $\theta_{norm}^{\left(s+\text{1}\right)}$. In this way, each parameter is updated with the most recent value of the other parameter, in turn. For non-standard conditional distributions, it may be necessary to use the Metropolis-Hastings algorithm for the purpose of more efficient sampling.

**Algorithm 1. Pseudocode of Bayesian SEM Estimation using Gibbs Sampling**

1 **Input:** Y, X, Λ, B, Γ, Ψ, α, $\theta_{norm}$,$\theta_{IW}$

2 for iteration in 1 to s:

3  $\eta_{new}=update\;\eta (Y,\;X,\;\Lambda ,\;B,\;\Gamma ,\;\Psi ,\;\alpha )$

4  $\theta_{norm_{new}}$ =$update\;\theta_{norm}(\eta_{new},\;\theta_{norm})$

5  $\theta_{IW_{new}}=update\;\theta_{IW}\left(\eta_{new},\theta_{IW}\right)$

6   $\alpha =update\;\alpha \left(\eta_{new},\alpha \right)$

7   $\Lambda =update\;\Lambda \left(\eta_{new},\Lambda \right)$

8   $B=update\;B\left(\eta_{new},B\right)$

9   $\Gamma =update\;\Gamma \left(\eta_{new},\Gamma \right)$

10  $\Psi =update\;\Psi \left(\eta_{new},\Psi \right)$

11  if not is conjugate $\left(\theta_{norm_{new}},\;\theta_{IW_{new}}\right)$:

12   $\theta_{norm_{new}},\theta_{IW_{new}}=metropolis-hastingsupdate\left(\theta_{norm_{new}},\theta_{IW_{new}}\right)$

13  store samples$\left(\eta_{new},\theta_{norm_{new}},\theta_{IW_{new}}\right)$

14 **Output:** posterior samples$\left(\eta_{new},\theta_{norm_{new}},\theta_{IW_{new}}\right)$

### 3.2. Direct and indirect effects in mediation analysis

**Fig 2:**
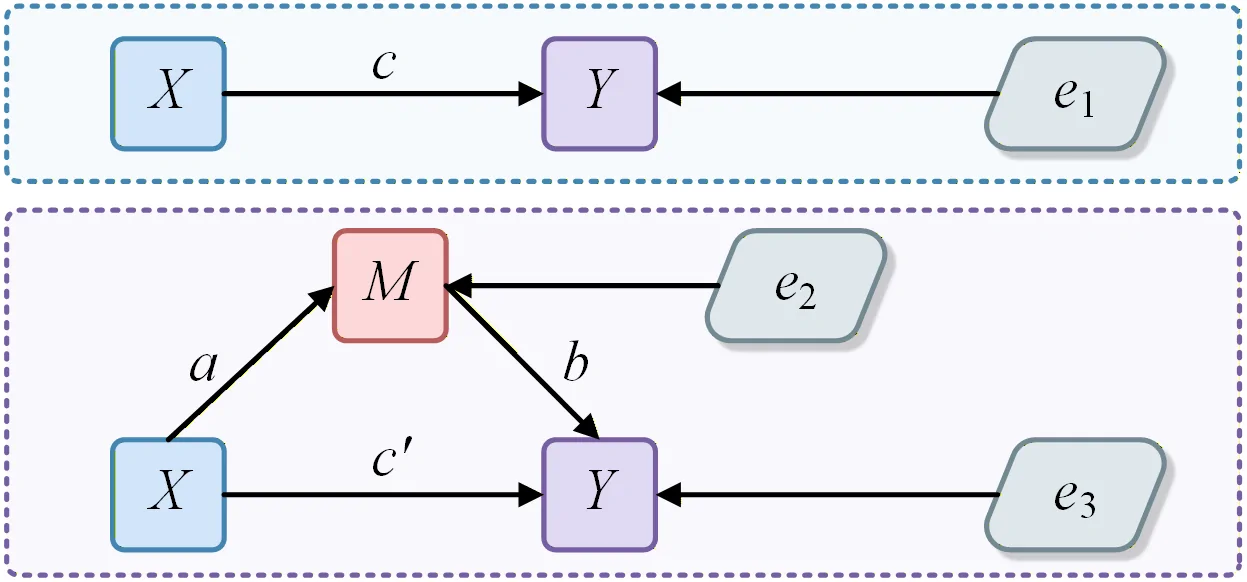
Classical mediation effects model.

There are two types of mediation effect indicators. One occurs in the interaction scenario, and the other occurs in the no-interaction scenario. Fig 2 displays the structure of classical mediation effect model.

Equations 7, 8, and 9 define the standard mediating effects model among the mediating effects. When the independent variable is X, the mediator is M, and the outcome variable is Y. The letters c, a, b, and c` stand for the estimations of the pertinent regression coefficients, where c indicates the overall impact of the independent variable X on the result Y variable.


$$\begin{array}{c} E\left(Y|\text{X}=\text{x}\right)=i+\text{cx}+\text{e1} \end{array}$$ (7)

$$\begin{array}{c} E\left(M|\text{X}=\text{x}\right)=i+\text{ax}+\text{e2} \end{array}$$ (8)

$$\begin{array}{c} E\left(Y|\text{X}=\text{x},\text{M}=\text{m}\right)=i+\text{c}`\text{x}+\text{bm}+\text{e3} \end{array}$$ (9)

Equations 7 and 9 serve as the foundation for the coefficient difference method’s definition of mediating effects. While c is the total effect of X on Y, c` is the influence of X net of the effect of the mediating variable. Given that the mediating variable indirectly affects the mechanism by which the independent variable X affects the result variable Y. Thus, IE=c−c`is the definition of the IE mediated by the mediator variable.

Equations 8 and 9, or two segments on the path from X→M→Y, serve as the foundation for the product of coefficients rule, which defines the indirect impact. The product of the coefficient abis the indirect effect. The default $\hat{a}\hat{b}$ distribution is asymmetrically skewed and matches the product distribution of two normally distributed random variables. There are two steps in the product distribution process. Using Equations 10 and 11, the point estimate A of the mediating effect is first determined by computing $\hat{a}$, $\hat{b}$, $\hat{\sigma }a$(standard error of a), and $\hat{\sigma }b$ (standard error of b). Second, the values of $\hat{a}$, $\hat{b}$, $\hat{\sigma }a$ (standard error of a), and $\hat{\sigma }b$ (standard error of b) acquired in the first stage are used to create confidence intervals.

According to the counterfactual-based paradigm, the mediating effect is defined as the existence of an interaction term between the mediator and the exposure in the outcome regression. Equation 10 can be utilized to compute the counterfactual outcome.


$$\begin{array}{c} E\left[Y\left(x,\text{M}(x*)\right)\right]=\Sigma_{m}E\left(Y|X=x,M=m\right)\left(M=m|X=x*\right) \end{array}$$ (10)

where x and $x^{*}$ represent the independent variable’s two distinct levels, respectively. Equation 11 represents the mediator regression model, assuming that the mediator and terminating variables are both variables that are continuous.


$$\begin{array}{c} E\left(M|X=x\right)=\beta_{\text{0}}+\alpha x+e_{\text{1}} \end{array}$$ (11)

Equation 12 is used to model the interaction between the mediating and independent variables.


$$\begin{array}{c} E\left(Y|X=x,M=m\right)=\beta_{\text{1}}+c`x+\beta_{\text{2}}m+e_{\text{2}} \end{array}$$ (12)

The counterfactual framework then defines three distinct effect values. where the independent variable level $X=x^{*}$ is compared with X=x for the result as indicated in Equation 13, and the controlled natural direct effect is represented as fixed M=m.


$$\begin{array}{c} CDE(m)=E\left(Y(x,m)\right)-E\left(Y(x*,m)\right) \end{array}$$ (13)

The NDE, fixing $M=M\left(x^{*}\right)$, and independent variable levels X=xand $X=x^{*}$ were used to compare the results, as shown in Equation 14.


$$\begin{array}{c} NDE=E\left(Y\left(x,M(x*)\right)\right)-E\left(Y\left(x*,M(x*)\right)\right) \end{array}$$ (14)

Equation 15 compares the outcomes using the NIE, with X=1, M=M(x), and $M=M\left(x^{*}\right)$, fixed.


$$\begin{array}{c} NIE=E\left(Y\left(x,M(x)\right)\right)-E\left(Y\left(x,M(x*)\right)\right) \end{array}$$ (15)

Equation 16 can then be used to break down the entire causal effect.


$$\begin{array}{c} TCE=E\left(Y(x)\right)-E\left(Y(x*)\right)=NDE+NIE \end{array}$$ (16)

The counterfactual theory allows for the definition of controls CDE, NDE, and NIE. The range of exposure is x* to x. The results are shown in Equation 17, Equation 18 and Equation 19, respectively.


$$\begin{array}{c} CDE(m)=\left(c`+\beta_{\text{3}}m\right)(x-x*) \end{array}$$ (17)

$$\begin{array}{c} NDE=\left(c`+\beta_{\text{0}}\beta_{\text{3}}+\beta_{\text{3}}ax*\right)(x-x*) \end{array}$$ (18)

$$\begin{array}{c} NIE=\left(a\beta_{\text{2}}+\beta_{\text{3}}ax\right)(x-x*) \end{array}$$ (19)

Both the direct and natural direct effects of the control are equal to $c`\left(x-x^{*}\right)$ in the absence of interactions $\left(\beta_{\text{3}}=\text{0}\right)$, while the NIE is equal to $a\beta_{\text{2}}(x-x*)$. This is in line with the effect’s value, ab, as described by the traditional regression approach.

The counterfactual framework provides the theoretical foundation for decomposing total effects into natural direct effects (NDE) and natural indirect effects (NIE). In this study, these counterfactual definitions are conceptually aligned with the mediation pathways specified in the structural equation model.

Specifically, the indirect effect estimated in the SEM corresponds to the natural indirect effect (NIE), while the direct path represents the natural direct effect (NDE) under the assumption of no interaction. Although the counterfactual framework is not directly estimated through simulation-based intervention analysis, it serves as an interpretive framework that enhances the causal meaning of mediation effects within the Bayesian SEM context.

### 3.3. Mediation analysis with SEM

The representation of the measurement model is shown in Equation 20 and Equation 21.


$$\begin{array}{c} y=\wedge y\eta +\varepsilon \end{array}$$ (20)

$$\begin{array}{c} x=\wedge x\xi +\delta \end{array}$$ (21)

The structural model is represented as shown in Equation 22.


$$\begin{array}{c} \eta =B\eta +\Gamma \xi +\varsigma \end{array}$$ (22)

where the endogenous variable’s measurement equation is Equation 20. A vector of p endogenous latent variables is denoted by y, which is p×1. With m endogenous latent variables, η is a m×1 vector. The p×q factor loading matrix of y on η is denoted by Λy. A p×1 vector made up of p measurement errors is called ε. Equation 21 is the measurement equation for the exogenous variables. With q exogenous indicators, x is the q×1 vector. The n×1 vector with n exogenous variables is called ξ. The q×n factor loading matrix of x on ξ is denoted by Λx. A q×1 vector made up of q measurement mistakes is called δ. The m×m coefficient matrix, B, in Equation 22 describes how the endogenous latent variables η affect one another. The influence that the external latent variable ξ has on the endogenous latent variable η is described by the m×n coefficient matrix Γ. The m×1 residual vector is denoted by ς.

The modeling assumptions are as follows. The measurement equation error terms ε and δ have a mean value of zero. The structural equation’s residual term ς also has a mean value of zero. Neither ε nor δ correlates with the components η and ξ, and the error terms ε and δ do not correlate with each other. There is also no relationship between ξ, ε, δ, and the residual term ς.

The eight parameter matrices that make up a full structural equation model are Λy, Λx, B, Γ, Φ, Ψ, Θε, and Θδ. Among these, the measurement equation or structural equation already has Λy, Λx, B, and Γ. The latent variable ξ’s covariance matrix is denoted by Φ. The residual term ς’s covariance matrix is denoted by Φ. The symbols Θε and Θδ represent the covariance matrices of ε and δ, respectively. The covariance matrix of the (p+q)×1 vector (y`,x`)` composed of all the indicators can be obtained by first calculating the covariance matrices of y, x, and the covariance matrices between them. where the covariance matrices of x and y are shown in Equations 23 and 24, respectively.


$$\begin{array}{c} \Sigma xx(\theta )=\wedge x\Phi \wedge x`+\Theta \delta \end{array}$$ (23)

$$\begin{array}{c} \Sigma yy(\theta )=\wedge yE(\eta \eta `)\wedge y`+\Theta \varepsilon \end{array}$$ (24)

Equation 25 displays the covariance matrix of x and y.


$$\begin{array}{c} \Sigma yx(\theta )=E(yx`)=\wedge yB\Gamma \Phi \wedge x` \end{array}$$ (25)

The overall covariance matrix Σ is equal to Σ(θ) if the theoretical model is correct, meaning that Σ=Σ(θ). As a result, the model parameters determine the observed variables’ variance and covariance.

Second, certain laws for model identification are necessary for structural equation modeling. These laws are usually referred to as the MIMIC rule, the two-step rule, and the t-rule. In certain unique model situations, the MIMIC rule is applied. Here, the two-step rule and the t-rule are primarily introduced. According to the t-rule, t is the number of unknown parameters in the model, and t≤(p+q)(p+q+1)/2 must exist for the model to be identifiable.

In the first stage of the two-step process, external and endogenous variables are not distinguished for the measurement portion. Together, they create an authenticated factorial model. The structural equation portion should then be viewed as a causal model made up of observable variables. If the exogenous and endogenous variables are judged observable, the full model can be found if the outcomes of the first and second stages of discrimination can be differentiated.

Estimating the matrices in the aforementioned expressions is the first step in solving SEM. In SEM, a variety of parameter estimation techniques can be applied. The assessment of the degree to which the matrix ∑(θ) (all of the aforementioned matrix expressions) resembles the covariance matrix S of the sample data is known as parameter estimation in maximum likelihood (ML) parameter estimation. The ML estimation allows the fitting function to minimize the estimate θ, as shown in Equation 26.


$$\begin{array}{c} FML=log|\Sigma \theta |-log|S|+\text{tr}\left(S\Sigma^{-\text{1}}(\theta )\right)-(p+q) \end{array}$$ (26)

where log|Σθ| and log|S| are the logarithms of the covariance matrices Σθ and S, respectively, and the determinant of those matrices. The symbol S represents the sum of the diagonals of the matrix $S\Sigma^{-\text{1}}(\theta )$. where the symbol (p+q) stands for all observed variables, or apparent variables in the measurement and structural models. The fitting function FML is altered by the logarithm of the great likelihood function. Therefore, it is necessary to assume that the indicator vectors have a multivariate normal distribution. The estimation that minimizes the fitting function FML is known as the great likelihood estimation.

Equation 27 displays the fitting function for the ULS approach.


$$\begin{array}{c} FULS=\frac{\text{1}}{\text{2}}tr\left[\left({S-\Sigma (\theta )}^{\text{2}}\right)\right] \end{array}$$ (27)

where the sum of squares for each element in the residual (residual) matrix ${S-\Sigma (\theta )}^{\text{2}}$ is equal to $tr\left[\left({S-\Sigma (\theta )}^{\text{2}}\right)\right]$. The weighted least squares estimate, which minimizes the FULS, has the benefit of being simple to comprehend. To make the covariance matrix Σ(θ) created from the model very close to the sample covariance matrix S, it is apparent to make each member of Σ(θ) very minuscule in difference from its S counterpart. Consequently, the distance of Σ(θ) from S is equal to the total of the squares of the differences of all elements. Unlike ML estimation, ULS estimation is not asymptotically efficient and does not require that the indicator has a normal distribution.

The estimation of these courses of action, X → M, Y → X, and M → Y, will be done concurrently instead of using regression to fit the path analysis of the trivariate model while looking at the mediation analysis. The instructions of Equation 28 are adhered to in this structural equation model.


$$\begin{array}{c} Y=\Gamma X+BY+\Psi \end{array}$$ (28)

Ycontains both M and Y, which are known as endogenous variables in the equations, whereas M is known as the exogenous variable. The mediation analysis’s primary focus is on the structural parameters Γ and B. Equation 29 and Equation 30 illustrate what these matrices have.


$$\begin{array}{c} \Gamma =[\begin{array}{c} \gamma_{MX}\\ \gamma_{YX} \end{array}] \end{array}$$ (29)

$$\begin{array}{c} B=[\begin{array}{cc} \text{0} & \text{0}\\ \beta_{YX} & \text{0} \end{array}] \end{array}$$ (30)

The exogenous variables make up the columns of matrixΓ, and they have an impact of $\gamma_{MX}$ and $\gamma_{YX}$ on the endogenous variables M and Y on the rows, respectively. The $\beta_{YX}$ term must be estimated. It stands for the Y → X action path. However the majority of the matrix B is made up of zeros, which represent unestimated action routes, such as M → Y. The entries in the matrices Γ and B will shortly be thoroughly explained via SEM. Notably, the matrix Ψ is also calculated, indicating the lack of fit of the endogenous model, or the values of 1-R2 when attempting to predict M and Y, as Equation 31 illustrates.


$$\begin{array}{c} \Psi =[\begin{array}{cc} \psi_{M} & \text{0}\\ \text{0} & \psi_{Y} \end{array}] \end{array}$$ (31)

Equation 32 is obtained by substituting equation 8 into equation 9, as seen in the mediator model schematic in Fig 2.


$$\begin{array}{c} Y=\text{c}`\text{X}+\text{b}\left(aX+e_{\text{2}}\right)+e_{\text{3}}=(c`+\text{ab})X+be_{\text{2}}+e_{\text{3}} \end{array}$$ (32)

A comparison of equations 32 and 9 leads to c=c`+ab. where the overall effect was broken down into a direct effect (c~) and a mediating effect (ab). The NIE is the mediating effect value utilized in this investigation. The multiplicative distribution approach and the asymmetric confidence interval method can all be used to perform the mediating effect test.

## 4. Experiment

### 4.1. Datasets and experimental setup

The data used in this study were collected from publicly available official sources, including the China Energy Construction database, the International Energy Agency (IEA), the General Administration of Customs of China, and the National Energy Administration. The observation period covers (2022–2023), which corresponds to the most recent years for which consistent and comparable data across all sources are available. The final dataset consists of 1,982 observations, where each observation corresponds to a country–year unit. Policy documents were collected at the national level and assigned to each country–year based on publication date and policy relevance. This cross-national panel structure allows for comparative analysis while maintaining consistency in data construction.

Data collection followed a standardized multi-step procedure. First, raw statistical data on energy trade volumes, investment activities, and cooperation projects were collected on an annual basis. Second, policy-related textual data, such as cooperation agreements, policy documents, and official statements, were retrieved from the same period. Third, all textual materials were cleaned and preprocessed through keyword screening and normalization. To operationalize multidimensional policy constructs, this study employs the TF-IDF method as a text-mining tool to quantify the relative importance of keywords within policy documents. Specifically, TF-IDF scores are used to transform qualitative policy information into quantitative indicators corresponding to predefined dimensions such as energy security, economic interests, environmental protection, and technological complementarity. It should be noted that TF-IDF is not part of the structural equation modeling estimation process itself. Instead, it serves as a data preprocessing and feature construction technique, generating observable indicators that are subsequently used to construct latent variables through exploratory and confirmatory factor analysis. These latent variables then enter the Bayesian SEM framework for mediation analysis. Finally, the resulting indicators were aggregated to construct multidimensional latent variables for policy preferences and energy cooperation, which served as inputs for subsequent factor analysis and mediation modeling. All steps rely on publicly accessible data and documented procedures, ensuring that the data construction process is transparent and reproducible.

The dependent variables of this paper, which are mainly energy trade and investment cooperation to establish the data. Finally, three dimensions of sub-dependent variables are established, which are the volume of energy trade, the number of investments, and the number of energy cooperation projects. The mediating variables are policy preferences. It is further subdivided into four sub-dimensions, namely energy security, economic interests, environmental protection, and technological complementarity.

First, keywords under different sub-dimensions were established, and five keywords with high representativeness and TF-IDF scores were selected, respectively. In the energy security dimension, keywords reflecting the country’s concern for energy supply stability and autonomous control ability are selected, including energy security, supply security, autonomous control, supply-demand balance, and security cooperation, emphasizing the country’s attention to energy risk management and supply security. In the economic benefits dimension, the keywords cover return on investment, trade benefits, cost-effectiveness, economic growth, and cooperation benefits, which focus on the importance of energy cooperation in promoting economic benefits and trade exchanges. Under the environmental protection dimension, the keywords focus on sustainable development and clean energy cooperation, including renewable energy, clean energy, carbon emission reduction, sustainable development, and green development, reflecting the concern of the partners for environmental responsibility and green transformation. Finally, the technology complementarity dimension selects keywords such as technology transfer, technology cooperation, innovation and R&D, technology exchange, energy science, and technology. The trend of innovation synergy and capacity enhancement through technological complementarity in energy cooperation is emphasized.

Then, the TF-IDF technique is used to measure the importance of words categorized in sub-dimensions in a text. A higher TF-IDF indicates that the word appears more in this text but less in the whole corpus and hence is important for this paper.

The independent variable of this paper, is chosen to be Policy and Regulatory Framework. It refers to the cooperation protocols, policy disciplines, regulatory arrangements, and enforcement systems set by countries in energy cooperation. It includes but is not limited to cooperation terms, information-sharing mechanisms, dispute resolution systems, etc., reflecting the role of the institutional level in guiding the willingness and effectiveness of cooperation. Similarly, the TF-IDF technique is used to capture and score high-frequency keywords, such as cooperation meetings and regulatory mechanisms.

In the experiments of this paper, Bayesian SEM analysis was done using the Mplus 8.2 demo version. The Bayesian Regression model was implemented by calling WinBUGS through the R2WinBUGS package in R 3.5.3 software. In addition, classical frequency school models were fitted using the proc calis and proc causalmed procedures of SAS 9.4, respectively. The total number of iterations was set to 20,000 for all Bayesian analyses, and the first 10,000 iterations were discarded as a burn-in period to obtain the posterior distributions of the parameters through continuous iterations. The convergence of the model is judged by the EPSR (potential reduction factor) and the posterior iteration plot (type = plot2 in Mplus). The results show that smooth convergence has been achieved for all key parameters. The final output includes the posterior estimates of the parameters, standard deviations, and 95% confidence intervals (CI), which are used for the comparative analysis of multiple methods. The thinning interval was set to 1 (default), as the convergence was excellent and the effective sample size was sufficient for inference.

During the model refinement process, several preliminary indicators were removed to improve the psychometric quality of the measurement model. Indicator retention was based on established SEM criteria rather than model fit optimization alone. Specifically, indicators exhibiting factor loadings below 0.60, substantial cross-loadings (greater than 0.40), or consistently large standardized residuals were considered for removal. Each deletion was evaluated together with its theoretical relevance to ensure that the conceptual meaning of the latent construct was preserved. Consequently, the final measurement model retained only indicators that satisfied both statistical and theoretical requirements, resulting in improved construct validity, discriminant validity, and overall model stability. This refinement procedure follows commonly accepted practices in structural equation modeling and enhances the transparency and reproducibility of the model development process.

### 4.2. Exploratory factor analysis (EFA)

Prior to conducting exploratory factor analysis, the adequacy of the sample size was evaluated. The sample size in this study meets commonly recommended criteria for factor analysis, including an acceptable ratio of observations to variables and an absolute sample size sufficient for stable factor extraction. Previous methodological studies suggest that factor analysis can yield reliable results when the subject-to-variable ratio exceeds conventional thresholds, particularly when communalities are high and factor loadings are strong.

In the present study, the high KMO values (>0.90), statistically significant Bartlett’s test of sphericity, and consistently high factor loadings jointly indicate that the data are suitable for factor analysis and that the extracted factor structure is robust.

SPSS 20.0 was used to conduct exploratory factor analysis on the two latent variable dimensions of policy preference and energy cooperation. The number of common factors based on the characteristic heel greater than or equal to one was ascertained using principal component analysis, which is based on principal component modeling. The reliability of the scale was also tested to evaluate the appropriateness of the scale to determine the degree of association between the dimensions and measures of the scale.

Exploratory factor analysis showed a KMO value of 0.920, and Bartlett’s test of sphericity resulted in a $\chi^{\text{2}}$ value of 7473.549 (with 1275 degrees of freedom, p < 0.0001). The exploratory factor analysis was conducted on an extended set of indicators, which included additional preliminary variables prior to model refinement. The reported Bartlett test degrees of freedom reflect this initial variable set. After factor screening and item reduction, the final model retained 20 items across four factors. This is appropriate for factor analysis since it shows that the correlation matrices of the data sets share similar factors and is statistically significant. The initial factor loading matrix was created by extracting the four common factors using principal component analysis. The rotated factor loading matrix was then obtained by oblique rotation. Four dimensions regarding policy preference were identified, and all extracted factors together explained 74.63% of the data variance, which reflects the representativeness of the factor analysis results. The exploratory factor loading values of the policy preference keywords are shown in Table 1, and all four selected dimensions consisted of three keywords with high TF-IDF scores. Especially, ES1, ES2, and ES3 represent keywords of energy safety including autonomous control, supply-demand balance, and security cooperation. EI1, EI2, and EI3 represent keywords of economic interests including cost-effectiveness, economic growth, and cooperation gains. EP1, EP2, and EP3 respectively represent the key terms of environmental protection including carbon reduction, sustainable development, and green development. TC1, TC2, and TC3 respectively represent the key terms of technological complementarity including innovative research and development, technological exchange, and energy technology.

**Table 1:** Results of exploratory factor analysis of policy preferences.

Number	Keyword	Factor	Loading
pp1	ES1	Energy safety	0.91
pp2	ES2		0.88
pp3	ES3		0.79
pp4	EI1	Economic interests	0.88
pp5	EI2		0.88
pp6	EI3		0.85
pp7	EP1	Environmental protection	0.87
pp8	EP2		0.85
pp9	EP3		0.84
pp10	TC1	Technological complementarity	0.85
pp11	TC2		0.83
pp12	TC3		0.77

**Fig 3:**
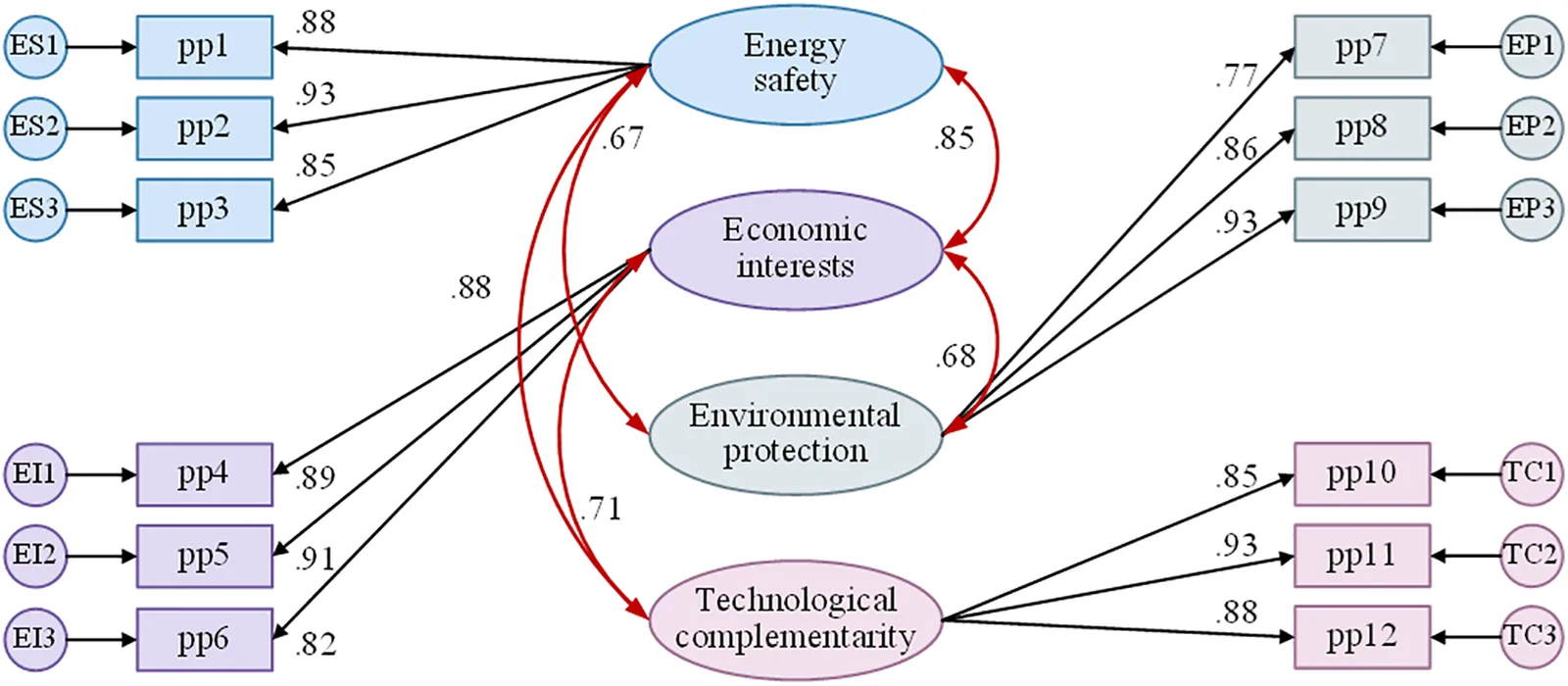
Standardized solution of the Confirmatory Factor Analysis (CFA) for Policy Preferences.

The results of the validated factor analysis for the policy preference keywords are shown in Fig 3, and the coefficients for each path are shown in Table 2. coefficients of unstandardized regression calculated using the greatest likelihood approach. In the model configuration, the measurement model results show that the observed indices of each latent variable exhibit good statistical properties. All factor loadings fall between 0.70 and 0.92, indicating that each index reflects its corresponding latent variable well while avoiding redundancy caused by excessive loading. The standard errors remain within a reasonable range, approximately 0.08–0.11, demonstrating high stability of the model estimation. Furthermore, the critical ratios for each path are significantly greater than 1.96, with corresponding p-values less than 0.001, indicating that all observed variables are statistically significantly loaded onto their corresponding latent variables, supporting the convergent validity of the measurement model.

**Table 2:** Table of path coefficients for policy preferences.

Indicator	Factor	Estimate	Std. Error	C.R.	P-value
pp2	Energy safety	1			
pp3		0.842	0.095	8.863	***
pp1		0.915	0.102	8.971	***
pp5	Economic interests	1			
pp6		0.801	0.097	8.257	***
pp4		0.876	0.105		***
pp7	Environmental protection	1			
pp8		0.784	0.091	8.615	***
pp9		0.859	0.094	9.138	***
pp10	Technological complementarity	1			
pp11		0.812	0.096	8.458	***
pp12		0.735	0.088	8.352	***

* Note: p-values with *** indicate that the probability value of significance is less than 0.001.

From the model fitting results, the overall model performance is good, which is shown in Table 3. RMSEA is 0.067, within the good range of 0.05–0.08, and SRMR is 0.054, below the recommended threshold of 0.08, indicating small residuals and a relatively ideal fit. Regarding augmented value fit indices, CFI of 0.937 and IFI of 0.934 are both significantly higher than 0.90, indicating that the model has stronger explanatory power compared to the independent model. The NFI of 0.912 and RFI of 0.903 are also at acceptable levels. Regarding parsimony fit indices, the PNFI of 0.642 and PCFI of 0.701 are both higher than 0.50, indicating that the model achieves a good balance between fit effectiveness and complexity. Furthermore, the AIC value is significantly lower than the comparison model, further supporting the model’s superiority.

**Table 3:** Overall fit test table for validated factor analysis of policy preference keywords.

Statistical Test	Standard/ Threshold	Result	Judgment
**Absolute Fit**			
RMSEA	<0.05 (excellent); 0.05–0.08 (good)	0.067	Good
SRMR	<0.08	0.054	Good
**Incremental Fit**			
NFI	>0.90	0.912	Acceptable
RFI	>0.90	0.903	Acceptable
IFI	>0.90	0.934	Good
CFI	>0.90	0.937	Good
**Parsimony Fit**			
PNFI	>0.50	0.642	Acceptable
PCFI	>0.50	0.701	Acceptable
AIC	Lower is better	2486 < 3494; 2486 < 8845	Good

Note: a represents the theoretical model value being less than the independent model value and also less than the saturated model value.

Table 4 displays the factor loading values of the entries. The exploratory factor loading values of the keywords for energy cooperation are shown in Table 4, and the selected dimensions are composed of keywords with high TF-IDF scores. Especially, Tr1, Tr2, and Tr3 represent the three keywords of energy trade volume including energy supply, import and export, and trade value. In1, In2 and In3 represent the keywords of number of investments including financial support, capital flows, and capital inflows. Co1, Co2, and Co3 represent the four keywords of number of cooperative projects including project implementation, joint development, and technology sharing.

**Table 4:** Results of exploratory factor analysis of energy cooperation.

Number	Keyword	Factor	Loading
ec1	Tr1	Energy trade volume	0.86
ec2	Tr2		0.79
ec3	Tr3		0.9
ec4	In1	Number of investments	0.82
ec5	In2		0.75
ec6	In3		0.67
ec7	Co1	Number of cooperative projects	0.89
ec8	Co2		0.85
ec9	Co3		0.94

**Fig 4:**
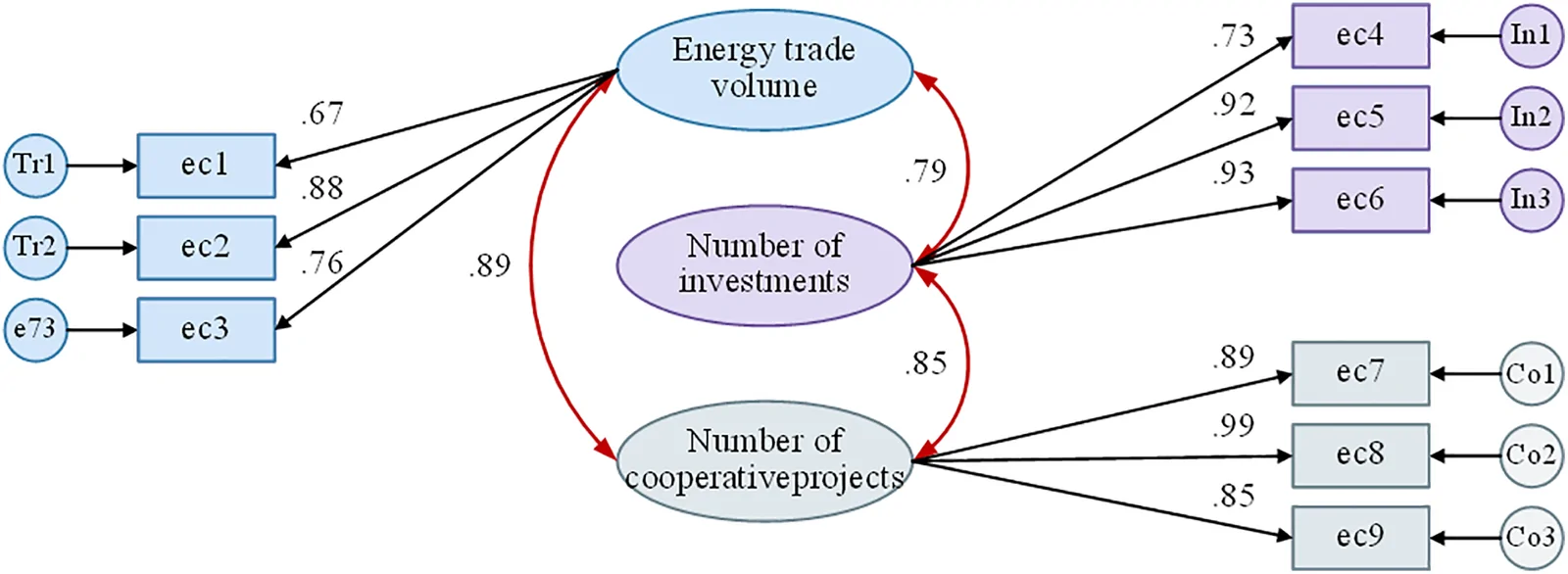
Confirmatory Factor Analysis (CFA) measurement model for Energy Cooperation Indicators.

Fig 4 displays the findings of the verified factor analysis of energy cooperation, while Table 5 displays the coefficients of each path. Overall, the loading coefficients of each observed indicator on their respective latent variables are all at a high level and have passed the significance test, indicating that the measurement model has good convergent validity. Specifically, in the dimension of energy trade volume, the standardized loading coefficients of each measurement indicator are all above 0.80, indicating that the relevant indicators can reflect the degree of energy trade cooperation well. In the dimension of investment quantity, the loading coefficients of each indicator are between 0.74 and 0.81, indicating that the variables related to investment cooperation have a strong explanatory power for the latent variables. In the dimension of the number of cooperation projects, the loading coefficients of each indicator are all above 0.82, indicating that the cooperation project indicator can stably characterize the depth of bilateral energy cooperation. Overall, the critical ratios (C.R.) of all measurement indicators are significantly higher than 1.96, and the significance level reaches 1%, indicating that the three-dimensional measurement structure of energy cooperation has high reliability and statistical stability. Table 6 displays the unstandardized regression coefficients that were generated using the great likelihood technique and the model’s fitness assessment. From the perspective of absolute fit indices, RMSEA is 0.068, below the recommended threshold of 0.08, indicating that the model residuals are within an acceptable range and the overall fit is good. SRMR is 0.056, also below the 0.08 standard, indicating that the difference between the sample covariance matrix and the theoretical model matrix is small. From the perspective of augmented fit indices, NFI, RFI, IFI, and CFI reach 0.912, 0.901, 0.928, and 0.931 respectively, all reaching or approaching the 0.90 standard, indicating that the current model has strong explanatory power compared to the independent model. From the perspective of parsimony fit indices, PNFI and PCFI are 0.668 and 0.726 respectively, both above 0.50, indicating that the model maintains good fit while possessing high structural parsimony. Furthermore, the AIC results show that the current model’s value is lower than that of the competing and saturated models, further validating the superiority of the model specification.

**Table 5:** Table of energy cooperation pathway coefficients.

Indicator	Factor	Estimate	Std. Error	C.R.	P-value
ec2	Energy trade volume	1	—	—	—
ec1		0.918	0.098	9.367	***
ec3		0.864	0.094	9.191	***
ec4	Number of investments	1	—	—	—
ec5		0.812	0.101	8.040	***
ec6		0.745	0.096	7.760	***
ec8	Number of cooperative projects	1	—	—	—
ec9		0.821	0.099	8.293	***
ec7		0.873	0.103	8.476	***

Note: p-value with *** indicates that the probability value of significance is less than 0.001.

**Table 6:** Overall fitness test table for validated factor analysis of energy cooperation.

Statistical test		Adapted standard or critical values	Data on test results	Model Adaptation Judgment
Absolute Fitness Index (AFI)	RMSEA SRMR	< 0.05 (excellent); 0.05-0.08 (good); <0.08	0.068 0.056	Good Good
Value Added Fitness Index	NFI	>0.90	0.912	Acceptable
	RFI	>0.90	0.901	Acceptable
	IFI	>0.90	0.928	Good
	CFI	>0.90	0.931	Good
Simplicity Fitness Index	PNFI	>0.50	0.668	Acceptable
	PCFI	>0.50	0.726	Acceptable
	AIC	Lower is better	520 < 557; 520 < 2449	Good

Note: a represents the theoretical model value being less than the independent model value and also less than the saturated model value.

Table 7 displays the correlation analysis between the latent variables of the keywords and the observed data based on the Pearson correlation coefficient’s magnitude. In Table 7 and Table 8, ES denotes Energy Safety, EI denotes Economic Interests, EP denotes Environmental Protection, TC denotes Technological Complementarity, TR denotes Energy trade volume, IN denotes Number of investments, CO denotes Number of Cooperative Projects, and PR denotes Policy & Regulatory Framework. The descriptive statistics show that the means of each variable are within a reasonable range, and the standard deviation is moderate, indicating that the sample data has a certain degree of dispersion and discriminative power, which can meet the needs of subsequent empirical analysis. The correlation analysis shows a significant positive correlation between ES and energy cooperation (Y), indicating that the higher the demand for energy security, the higher the level of bilateral energy cooperation. This suggests that energy supply stability, supply chain security, and resource security needs are important factors driving cooperation. EI also show a significant positive correlation with energy cooperation, indicating that trade gains, investment returns, and market expansion expectations can effectively promote the deepening of cooperative relationships. EP also shows a positive relationship with energy cooperation, indicating that green transformation, low-carbon development goals, and environmental governance needs are becoming important new drivers for energy cooperation. A significant positive correlation exists between TC and energy cooperation, indicating that when both parties have strong complementarity in technological capabilities, industrial structure, and resource endowments, it is easier to form a stable cooperative relationship. This result shows that technological synergy and industrial collaboration are important conditions for improving the efficiency of energy cooperation. A significant positive correlation was also found between PR and energy cooperation, indicating that consistency in policy orientation, degree of institutional coordination, and convergence of strategic perceptions can effectively promote the improvement of cooperation levels.

**Table 7:** Relevant structural analysis.

	ES	EI	EP	TC	TR	IN	CO	PR
**ES**	1							
**EI**	0.532**	1						
**EP**	0.468**	0.689**	1					
**TC**	0.452**	0.703**	0.682**	1				
**TR**	0.684**	0.528**	0.472**	0.438**	1			
**IN**	0.623**	0.501**	0.463**	0.352**	0.712**	1		
**CO**	0.421**	0.681**	0.723**	0.689**	0.452**	0.398**	1	
**PR**	0.214*	0.298**	0.256**	0.287**	0.182*	0.176*	0.205**	1

Note: ** Correlation is significant at 0.01, * indicates correlation is significant at 0.05.

**Table 8:** Structural model path coefficient table.

From	To	Estimate	Standard error	Critical ratio	Significance P-value
PR	ES	0.412	0.104	3.962	***
	EI	0.238	0.058	4.103	***
	EP	0.265	0.061	4.344	***
	TC	0.192	0.053	3.623	***
	TR	0.118	0.067	1.761	0.078
	IN	0.102	0.062	1.645	0.100
	CO	0.136	0.058	2.345	0.019
ES	TR	0.284	0.143	1.986	0.047
	IN	0.236	0.128	1.842	0.066
	CO	0.198	0.121	1.636	0.102
EI	TR	0.214	0.116	1.845	0.065
	IN	0.172	0.102	1.686	0.092
	CO	0.301	0.098	3.071	**
EP	TR	0.512	0.118	4.339	***
	IN	0.498	0.107	4.654	***
	CO	0.643	0.095	6.768	***
TC	TR	0.205	0.098	2.092	0.036
	IN	0.144	0.087	1.655	0.098
	CO	0.176	0.081	2.173	0.030

Note: p-values with *** indicate that the probability value of significance is less than 0.001.

Furthermore, a moderate positive correlation was generally observed among the explanatory variables, suggesting that energy security, economic interests, environmental protection, and technological complementarity are not isolated entities, but rather mutually influential and synergistic in the actual process of cooperation.

**Fig 5:**
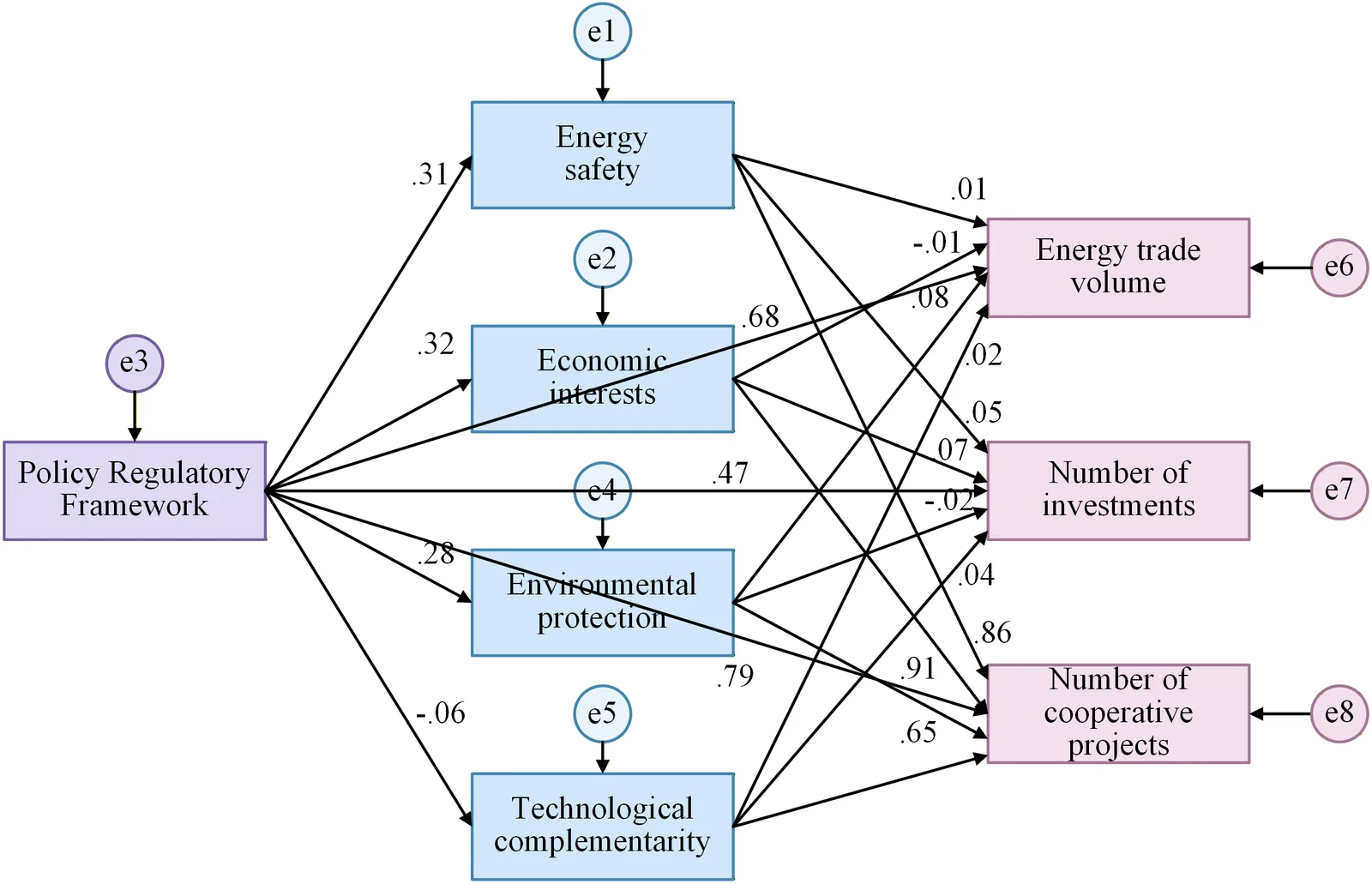
Path Diagram of the Structural Model for Policy Impact on Energy Cooperation.

The structural equation model was created using the previously discussed factor analysis and associated structural analysis, as illustrated in Fig 5. Table 8 displays the predicted model fit index and path coefficients. Table 9 displays the model’s fitness evaluation using the unstandardized regression coefficients that were calculated using the approach of great likelihood.

**Table 9:** Structural Model Overall Fit Checklist.

Statistical test		Adapted standard or critical values	Data on test results	Model Adaptation Judgment
Absolute Fitness Index (AFI)	RMSEA	<0.05 (excellent); 0.05–0.08 (good)	0.071	Y
Value Added Fitness Index	NFI	>0.90	**0.914**	Y
	RFI	>0.90	**0.903**	Y
	IFI	>0.90	**0.932**	Y
	CFI	>0.90	**0.936**	Y
Simplicity Fitness Index	PNFI	>0.50	**0.642**	Y
	PCFI	>0.50	**0.701**	Y
	AIC	Lower is better	**548 < 1540; 548 < 1600**	Y

Note: a represents the theoretical model value being less than the independent model value and also less than the saturated model value.

First, PR has a significant positive impact on ES, EI, and EP, indicating that policy orientation plays a crucial role in shaping national perceptions of energy cooperation. When policy coordination improves, countries are more inclined to strengthen energy security, expand the benefits of economic cooperation, and enhance cooperation in environmental governance. Among these, policy preferences have a relatively high impact coefficient on energy security, suggesting that security issues remain a core focus in energy cooperation. Second, regarding the variables affecting the outcomes of energy cooperation, EP shows the most significant positive effect, significantly promoting energy trade volume, investment scale, and the number of cooperative projects. This indicates that green development goals and the need for low-carbon transformation have become important driving forces for energy cooperation. This suggests that, in the current context of global energy transition, environmental governance cooperation is gradually shifting from a secondary issue to a core driver of energy cooperation. ES also shows a positive impact on all dimensions of energy cooperation. Although the significance of some pathways is relatively limited, the overall direction is in line with expectations, indicating that stable energy supply, resource security, and supply chain security remain important fundamental conditions for promoting cooperation. EI has a certain positive effect on energy cooperation, indicating that market returns, investment returns, and expectations of trade expansion remain important practical drivers for cooperation. TC has a positive impact on energy cooperation, indicating that when cooperating parties have complementary advantages in technological capabilities, industrial chain structure, and resource endowments, it is more conducive to forming a sustainable cooperative relationship.

Table 9 further reports the overall goodness-of-fit results of the structural model. From the perspective of absolute fit indices, RMSEA is within the acceptable range, indicating a low level of model residuals and a good match between the theoretical structure and the sample data. From the perspective of added-value fit indices, NFI, IFI, and CFI all meet or approach commonly recommended standards, indicating that the model constructed in this paper has stronger explanatory power compared to independent models. From the perspective of parsimony fit indices, PNFI and PCFI are both above 0.50, indicating that the model maintains good fit while possessing good structural parsimony. Furthermore, the AIC comparison results show that the current model is superior to the competing and saturated models, further supporting the rationality of the model specification.

### 4.3. Analysis of intermediation effects

In this study, the composite scores of the scales are used to construct a simple three-variable data structure, i.e., Policy and Regulatory Framework (X), Policy preference (M) and Energy cooperation (Y). These three variables are described and correlated in Table 10 below. The correlation coefficient results show a significant positive correlation between policy and institutional framework (X) and policy preference (M), indicating that the more complete institutional coordination and policy arrangements are, the more conducive it is to form a positive cooperative preference. A significant positive correlation also exists between policy preference (M) and energy cooperation (Y), suggesting that policy consistency and strategic consensus can effectively promote the improvement of energy cooperation levels. In contrast, the direct correlation between policy and institutional framework (X) and energy cooperation (Y) is relatively weak, indicating that its influence may be more effectively achieved through the mediating mechanism of policy preference.

**Table 10:** Means/percentages and correlation coefficients of the general conditions of the study population.

Variable	Mean ± SD	Correlation Coefficient		
		Y	M	X
Energy cooperation (Y)	86.24 ± 23.46	1.000	0.419**	0.238*
Policy preference (M)	73.65 ± 10.27	0.426**	1.000	0.312**
Policy & Regulatory Framework (X)	18.72 ± 3.89	0.245*	0.326**	1.000

Note: With * indicates p < 0.05, statistically significant.

Table 11 reports the results of mediating effect estimation under the counterfactual framework, used to examine the mechanism by which policy and institutional frameworks (X) influence energy cooperation (Y) through policy preferences (M). The results show that policy and institutional frameworks have a significant positive impact on policy preferences, and policy preferences further significantly promote energy cooperation, indicating that the mediating transmission path is valid.

**Table 11:** Simple mediation model results for Bayesian SEM estimation.

Variable		Estimate	Standard error	P-value	95% confidence interval
regression coefficient	Y and X	0.214	0.187	0.256	(−0.153, 0.581)
	Y and XM	0.012	0.018	0.238	(−0.023, 0.047)
	Y and M	0.643	0.214	0.004**	(0.224, 1.062)
	M and X	0.472	0.136	0.001***	(0.205, 0.739)
intercept	Intercept (Y)	87.421	24.118	0.002**	(40.160, 134.682)
	Intercept (M)	62.315	3.218	0.000***	(56.007, 68.623)
error term	Error term (Y)	196.542	21.317	0.000***	(154.760, 238.324)
	Error term (M)	92.418	10.214	0.000***	(72.397, 112.439)
efficiency value	TIE	0.305	0.121	0.012**	(0.068, 0.542)
	PIE	0.298	0.115	0.009**	(0.073, 0.523)
	DE	0.087	0.187	0.642	(−0.279, 0.453)

Note: With * indicates p < 0.05, statistically significant.

From the effect decomposition results, both the total effect (TIE) and the indirect effect (PIE) are positive and statistically significant, indicating that policy and institutional frameworks can effectively improve the level of energy cooperation through policy preferences. In contrast, the direct effect (DE) is not significant, suggesting that the influence of institutional factors on energy cooperation is mainly achieved through the mediating variable of policy preferences, rather than through direct action.

**Fig 6:**
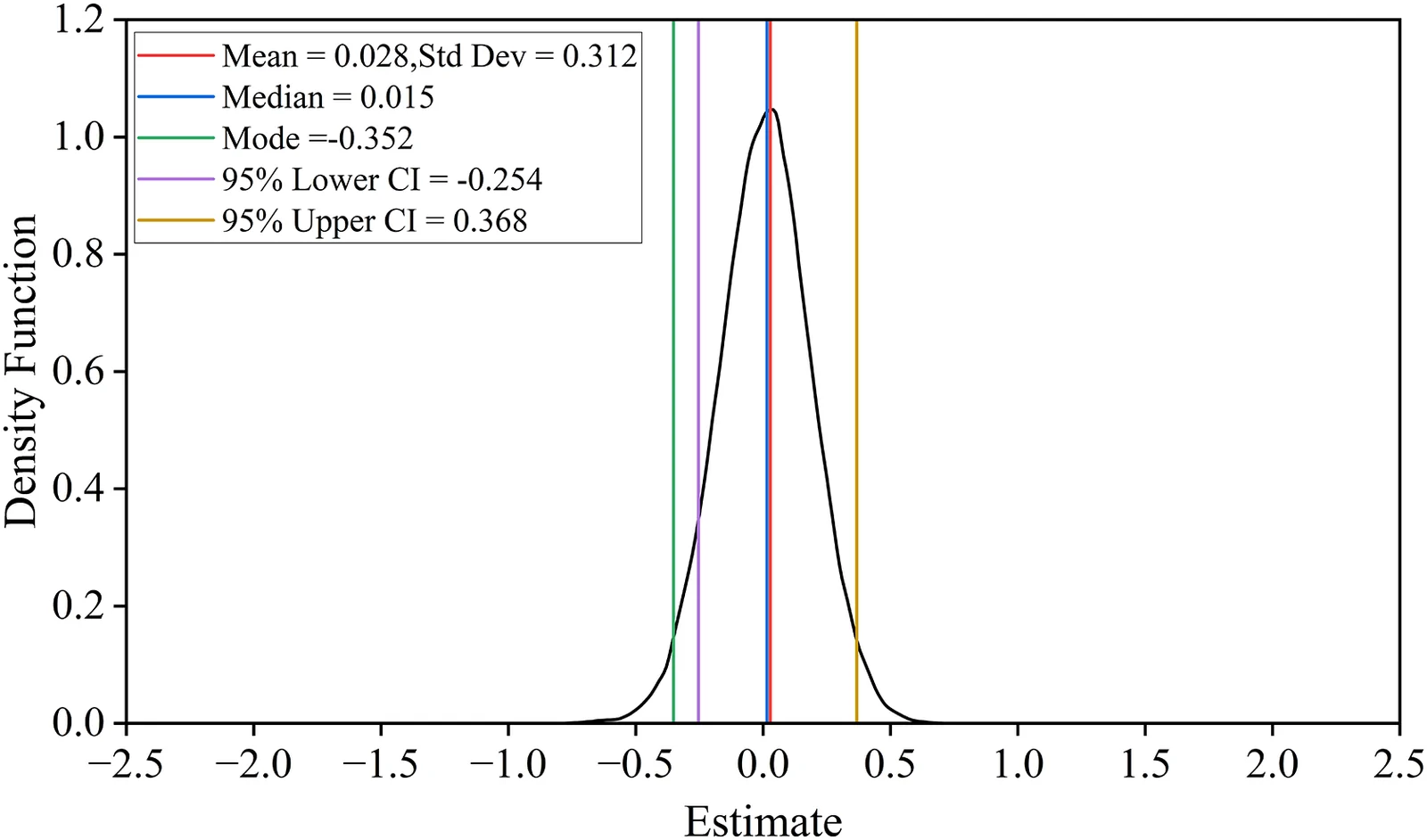
The Posterior density map of PIE.

**Fig 7:**
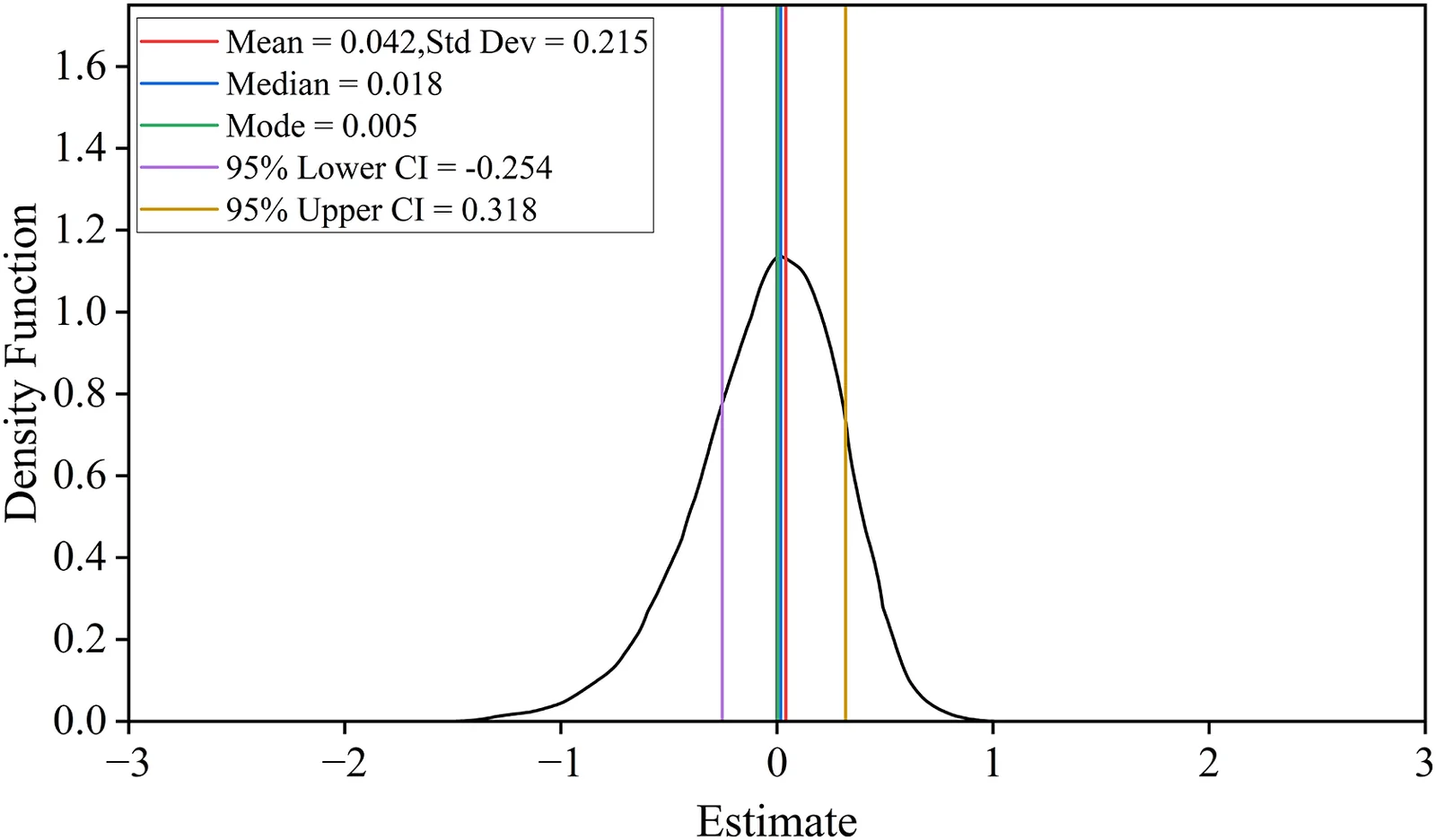
The Posterior density map of DE.

**Fig 8:**
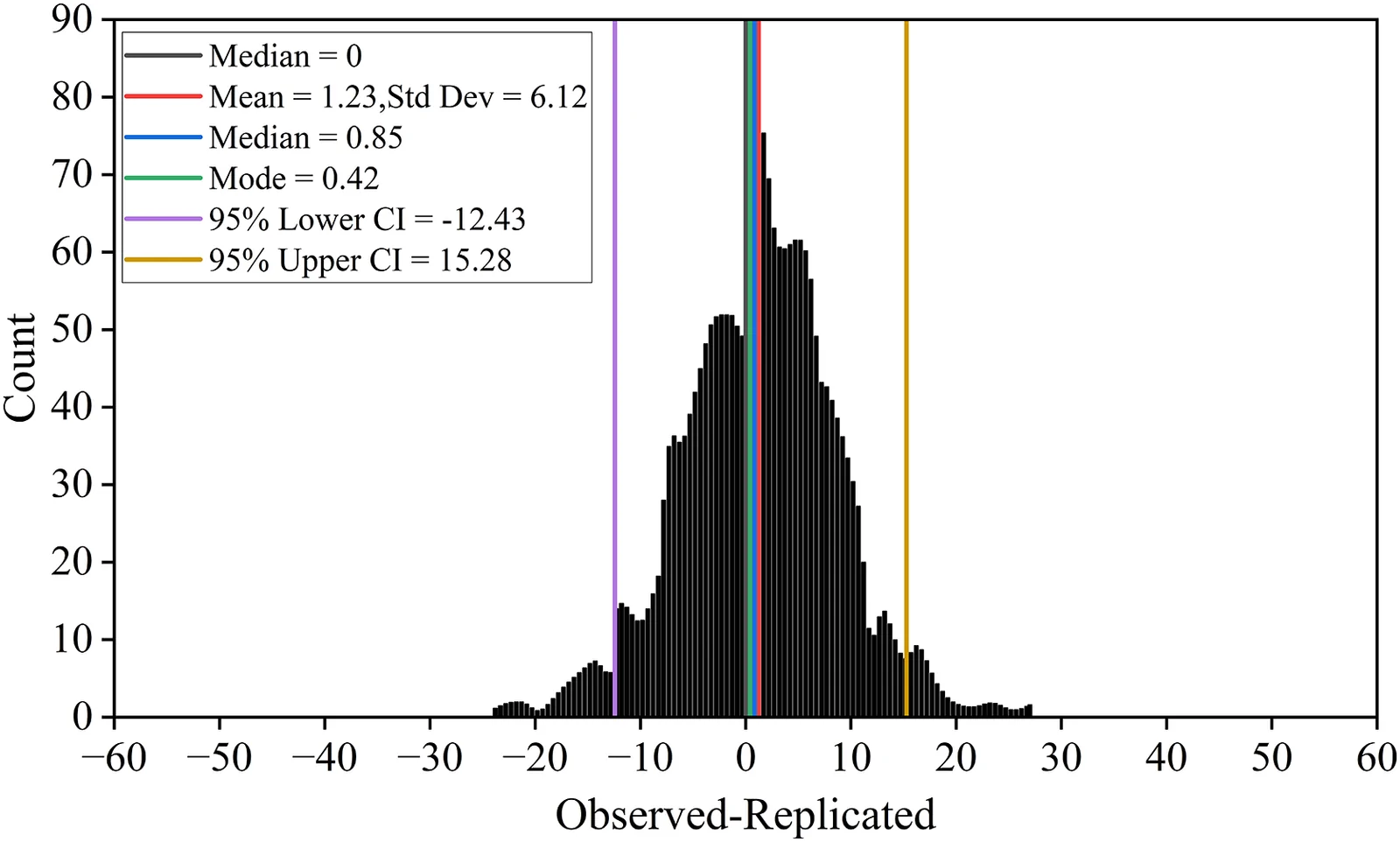
Histogram of observed-replicated differences for PPC.

**Fig 9:**
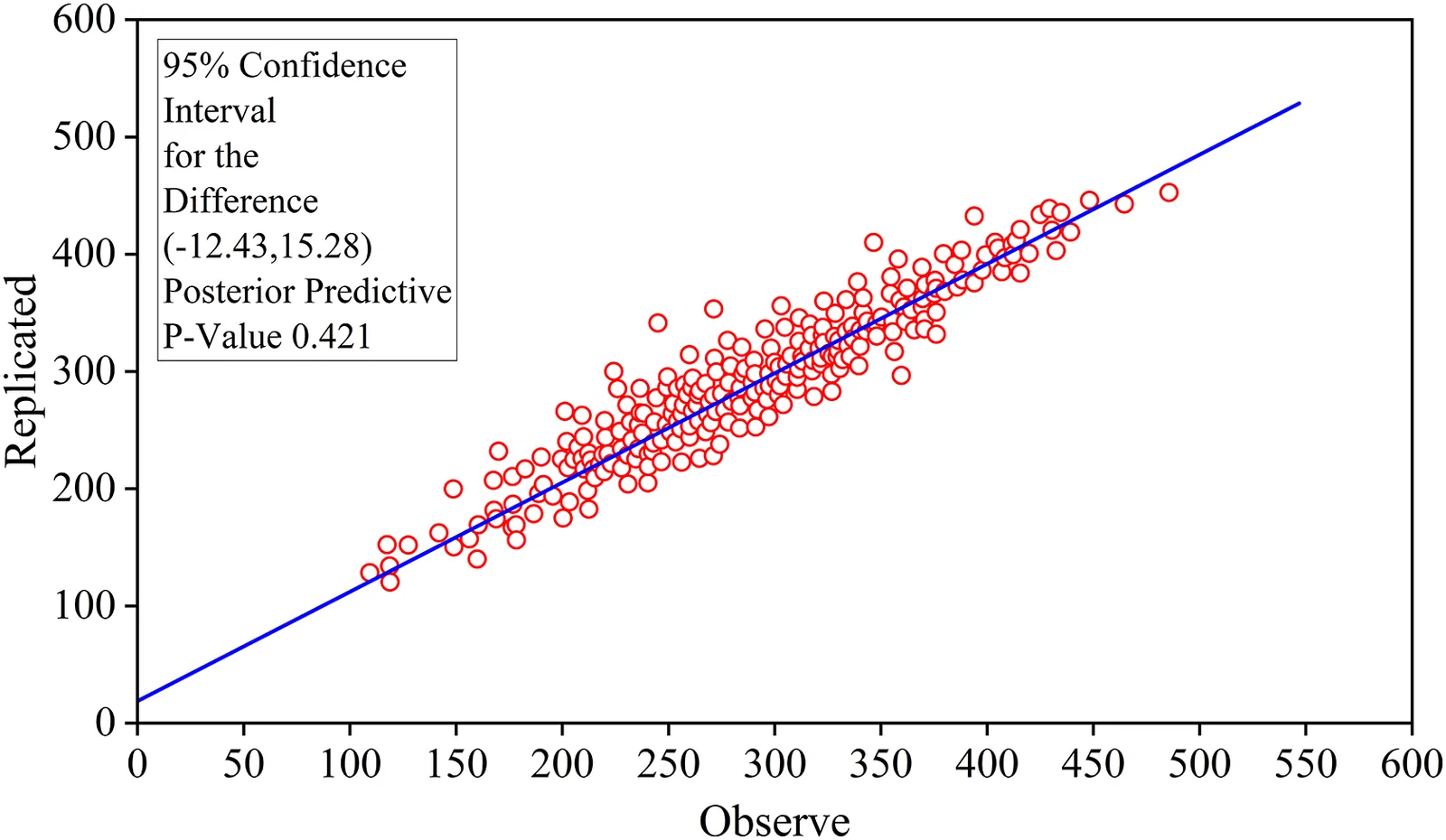
Scatterplot of Observed vs. Replicated Values.

Fig 6 shows the posterior density distribution of the Total Indirect Effect (TIE). Overall, the posterior distribution is unimodal and approximately normal, with a mean of 0.042, a median of 0.018, and a mode of 0.005, indicating relatively stable estimation results. The 95% confidence interval is (−0.254, 0.318), with a relatively concentrated range, indicating that the total effect estimation has a certain degree of robustness. In summary, Fig 6 illustrates that policies and institutional frameworks have a combined impact on energy cooperation through direct and indirect paths, but the overall effect magnitude is relatively mild. Fig 7 shows the posterior density distribution of the Direct Effect (DE). The results show that this distribution also exhibits a relatively smooth unimodal structure, with a mean of 0.028 and a median of 0.015, indicating that the direct effect is close to zero. Its 95% confidence interval is (−0.254, 0.368), covering the zero point, indicating that the direct impact of policies and institutional frameworks on energy cooperation is not significant. This means that institutional arrangements themselves do not directly and significantly enhance energy cooperation; their role is primarily achieved through intermediary mechanisms. Fig 8 shows the posterior prediction distribution of the difference between observed and replicated values, used to examine the model’s fit. The results show that the distribution generally revolves around zero, with a mean of 1.23, a median of 0.85, and a 95% confidence interval of (−12.43, 15.28), indicating a small difference between the model-generated data and the actual observed data, and no systematic bias. The approximately symmetrical distribution further demonstrates the model’s good predictive stability and estimation reliability. Fig 9 further provides a scatter plot of the posterior predictive check (PPC). Most sample points are distributed near the 45-degree reference line, indicating a high consistency between the model’s replicated values and the actual observed values. The posterior predictive P-value is 0.421, within a reasonable range, indicating that the model can reproduce the sample data structure well and there is no significant misfit problem. As shown in Fig 8, the Bayesian structural equation model exhibits good overall fit, and its specification demonstrates strong reliability.

Table 12 reports the mediation effect estimation results obtained using classical statistical methods, used for comparison with Bayesian estimation results and to test the robustness of the research conclusions. Overall, the results obtained by the classical method are basically consistent with the Bayesian estimation direction in Table 11, indicating that the mediation effect conclusions of this paper have high stability and consistency.

**Table 12:** Simple mediation model results for classical SEM estimation.

Variable		Estimate	Standard error	P-value	95% confidence interval
regression coefficient	Y and X	0.215	0.184	0.244	(−0.146, 0.576)
	Y and XM	0.011	0.019	0.566	(−0.026, 0.048)
	Y and M	0.624	0.207	0.006**	(0.218, 1.030)
	M and X	0.468	0.133	0.001***	(0.207, 0.729)
intercept	Y	87.392	24.081	0.002**	(40.193, 134.591)
	M	62.118	3.207	0.000***	(55.832, 68.404)
error term	Y	198.421	20.884	0.000***	(157.488, 239.354)
	M	93.764	9.986	0.000***	(74.192, 113.336)
efficiency value	TIE	0.292	0.118	0.014**	(0.061, 0.523)
	PIE	0.284	0.112	0.011**	(0.065, 0.503)
	DE	0.081	0.184	0.661	(−0.279, 0.441)

Specifically, the policy and institutional framework (X) still shows a significant positive impact on policy preference (M), indicating that the higher the degree of institutional coordination, the more conducive it is to forming a positive cooperative preference. At the same time, policy preference (M) also shows a significant positive effect on energy cooperation (Y), further verifying the mechanism of policy preference as a key mediating variable. From the effect decomposition results, both the total effect (TIE) and the indirect effect (PIE) reach statistical significance and the estimated values are positive, indicating that the policy and institutional framework can effectively promote energy cooperation through policy preference. In contrast, the direct effect (DE) does not reach a significant level, indicating that the influence of institutional factors on energy cooperation is mainly achieved through indirect paths rather than direct effects. This result is highly consistent with the Bayesian estimation results, further supporting the effect structure of complete mediation or mainly indirect effects.

Furthermore, the classical and Bayesian estimations show little difference in coefficient magnitude and significance, indicating that the conclusions of this study do not depend on a specific estimation method and possess good methodological robustness. This also demonstrates that, under different statistical frameworks, policy preferences are a crucial transmission mechanism connecting institutional arrangements and energy cooperation.

### 4.4. Comparison with mediation effect analysis of different models

To further test the robustness of the research findings, this paper compares the natural indirect effects (NIE) using various estimation methods, including Classical Structural Equation Modeling (SEM), Bayesian regression, classical regression mediation model, Generalized Structural Equation Modeling (GSEM), counterfactual mediation analysis, mixed effects model, GEE model, PLS-SEM, SUR model, dual machine learning (DML), TMLE, and Bayesian SEM. Table 13 shows the estimation results of different methods. Overall, the estimated mediation effects obtained by each model are all positive and statistically significant, indicating that policy preferences have a stable and significant mediating role between institutional frameworks and energy cooperation.

**Table 13:** Comparison of the NIE of the four methods.

Estimation method	Estimate	Standard error	P-value	95% confidence interval
Classical SEM	0.312	0.118	0.004	(0.084, 0.547)
Bayesian regression	0.284	0.105	0.007	(0.081, 0.487)
Classical regression (mediation)	0.298	0.097	0.005	(0.108, 0.492)
GSEM (GLM-based SEM)	0.306	0.121	0.006	(0.073, 0.553)
Counterfactual mediation (IKT)	0.291	0.115	0.008	(0.064, 0.514)
Mixed-effects mediation	0.305	0.129	0.011	(0.052, 0.558)
GEE mediation	0.276	0.118	0.013	(0.041, 0.508)
PLS-SEM	0.289	0.112	0.007	(0.067, 0.513)
SUR + Delta/Bootstrap	0.297	0.120	0.005	(0.062, 0.534)
Double Machine Learning (DML) mediation	0.271	0.109	0.012	(0.048, 0.498)
TMLE mediation	0.283	0.114	0.009	(0.059, 0.509)
**Bayesian SEM**	**0.305**	**0.123**	**0.006**	**(0.061, 0.552)**

The estimation results show that the estimated NIE values obtained by each method range from 0.271 to 0.312, with relatively small fluctuations, indicating that different models have high consistency in identifying the mediation effects. Among the estimates, the classical structural equation model yielded the highest value (0.312), followed by the Bayesian structural equation model (0.305) and the generalized structural equation model (0.306). These results are relatively close, indicating that latent variable modeling can reliably identify the transmission path of institutional factors influencing energy cooperation through policy preferences. Meanwhile, the results obtained by dual machine learning (0.271), the GEE model (0.276), and TMLE (0.283) were slightly lower, but still significant, further demonstrating that the research conclusions do not depend on specific estimation techniques. These results indicate that policy and institutional frameworks do not directly and automatically translate into energy cooperation performance; their influence primarily manifests indirectly by shaping national policy preferences. In other words, institutional arrangements are essentially external governance conditions, and only when they promote policy coordination, enhance strategic mutual trust, and form common development goals can they effectively drive the achievement of cooperative outcomes. Theoretically, this finding suggests that international energy cooperation is not only constrained by formal institutions but also profoundly influenced by latent preference structures. Policy preferences, reflecting energy security demands, economic interests, environmental governance goals, and the need for technological synergy, form a crucial internal mechanism connecting institutional arrangements and cooperation performance.

## 5. Discussion

This study demonstrates that policy preferences constitute the primary mechanism through which institutional frameworks influence international energy cooperation. Although institutional arrangements establish the governance environment for cooperation, their direct effect on cooperation outcomes is relatively weak after accounting for the mediating role of policy preferences. This finding suggests that institutions function primarily as enabling mechanisms that shape national strategic priorities, policy coordination, and cooperation expectations rather than directly generating cooperative outcomes. In practice, differences in implementation capacity, political priorities, governance efficiency, and strategic objectives across countries often weaken the direct influence of formal institutional arrangements. Consequently, institutional advantages can only be translated into tangible cooperation outcomes when they are internalized into shared policy preferences and coordinated strategic actions.

The results further indicate that environmental protection and technological complementarity have become increasingly important dimensions of policy preferences driving international energy cooperation. While previous studies have primarily emphasized energy security and economic interests as the dominant motivations for cooperation, the present findings suggest that global carbon neutrality commitments, green transition strategies, and technological innovation are reshaping the logic of international energy partnerships. The growing importance of low-carbon development, clean energy technologies, and technological collaboration reflects the evolving policy priorities of participating countries and highlights the transition from resource-oriented cooperation toward sustainability-oriented cooperation.

From a methodological perspective, the comparative analyses demonstrate the advantages of Bayesian structural equation modeling for evaluating complex mediation mechanisms. Compared with conventional regression-based mediation analysis and frequentist SEM approaches, Bayesian SEM provides more stable parameter estimates, more reliable interval estimation, and greater robustness in handling latent-variable measurement error and parameter uncertainty. These advantages are particularly valuable for policy research involving multidimensional latent constructs, complex mediation structures, and moderate sample sizes. The consistency of the mediation estimates across multiple analytical approaches further strengthens confidence in the robustness of the proposed framework and supports the application of Bayesian SEM in future studies of policy evaluation, international cooperation, and energy governance.

An additional methodological implication concerns the sensitivity of structural equation modeling to measurement specification. The noticeable improvement in model fit following indicator refinement illustrates that the quality of latent variable measurement substantially influences parameter estimation and overall model performance. Because latent constructs are represented indirectly through observed indicators, weak or cross-loading items may introduce measurement error and reduce construct validity. In this study, indicator refinement was conducted according to predefined psychometric criteria—including factor loadings, cross-loadings, and residual diagnostics—together with theoretical considerations, rather than solely to optimize goodness-of-fit indices. This transparent refinement process ensured that the retained indicators adequately represented the conceptual meaning of each latent construct while improving measurement reliability and validity. Nevertheless, the observed sensitivity also suggests that future research should further validate the measurement framework using independent datasets, alternative policy contexts, and cross-validation procedures. Such efforts would enhance the generalizability and reproducibility of the proposed Bayesian SEM framework and contribute to the development of more robust measurement systems for policy preference analysis.

In the initial measurement model, each latent construct was represented by a relatively large number of observed indicators. Although this specification was intended to comprehensively capture the underlying dimensions, several indicators were conceptually overlapping and exhibited extremely high standardized factor loadings (greater than 0.95). Such results suggested substantial redundancy among the observed variables, insufficient discriminant validity, and potential overfitting of the measurement model. Consequently, the original CFA and SEM demonstrated poor model fit, with unacceptable fit indices (e.g., RMSEA = 0.399 and CFI = 0.577), unstable structural path estimates, and posterior predictive diagnostics indicating considerable discrepancies between the model and the observed data. These findings suggested that the original measurement structure could not adequately represent the latent constructs and required further refinement.

To address these limitations, the measurement model was systematically reconstructed. First, conceptually redundant indicators were identified through theoretical evaluation, TF-IDF relevance analysis, cross-loading assessment, and factor loading diagnostics. Indicators that provided overlapping information or exhibited excessively high loadings were removed, while the most representative and statistically stable indicators were retained for each latent construct. As a result, the number of observed indicators for each policy preference construct was reduced from five to three, yielding a more parsimonious and theoretically coherent measurement framework. Following this optimization, all exploratory factor analysis (EFA), confirmatory factor analysis (CFA), Bayesian structural equation modeling (SEM), and mediation analyses were re-estimated using the revised indicator set. The revised measurement model demonstrated substantial improvements in both statistical performance and construct validity. Standardized factor loadings were reduced to an appropriate range (approximately 0.77–0.91), indicating improved discriminant validity and reduced redundancy among indicators. Model fit indices also improved markedly, with the revised CFA and SEM achieving acceptable fit (e.g., RMSEA = 0.067 for the measurement model and RMSEA = 0.074 for the structural model, together with CFI values above 0.90). Furthermore, the revised posterior predictive diagnostics showed much closer agreement between the observed and replicated data, and Bayesian and classical estimation results remained directionally consistent while no longer producing identical parameter estimates. These improvements indicate that the revised model provides a more reliable, parsimonious, and theoretically meaningful representation of the relationships among institutional frameworks, policy preferences, and energy cooperation.

Overall, the findings consistently support the central conclusion that policy preferences serve as the key transmission mechanism linking institutional frameworks to energy cooperation outcomes. By integrating text-based policy measurement with Bayesian structural equation modeling, this study provides both a substantive explanation of how institutional effects are translated into international energy cooperation and a methodological framework capable of modeling latent policy processes under uncertainty. These contributions not only deepen the understanding of the institutional foundations of international energy cooperation but also provide a rigorous analytical framework that can be extended to broader studies of policy implementation, international governance, and sustainable energy transition.

## 6. Conclusion

This paper developed an integrated analytical framework combining text quantification, latent variable modeling, and mediation analysis to examine how policy preferences influence energy cooperation through indirect pathways. Using the TF-IDF method, a multidimensional indicator system was constructed, followed by EFA and CFA to verify measurement validity, and SEM was employed to estimate direct and indirect effects. The results show that policy preferences play a significant mediating role between institutional frameworks and energy cooperation outcomes. Across 12 estimation methods, the natural indirect effects are consistently positive and statistically significant, with estimates ranging from 0.271 to 0.312. This indicates that institutional arrangements promote cooperation mainly by shaping policy preferences rather than through direct effects alone. Among the methods, Bayesian SEM performs particularly well in handling latent variables, parameter uncertainty, and measurement error, producing more stable estimates and reliable interval coverage. This suggests that Bayesian SEM is especially suitable for complex policy and cooperation research with relatively limited samples. From a policy perspective, improving institutional quality alone is insufficient unless accompanied by stronger policy alignment. Governments should therefore reduce mismatches in environmental standards, technological priorities, and regulatory expectations through shared frameworks, dialogue mechanisms, and joint planning. Strengthening technological complementarity and integrating environmental objectives as common interests can further enhance long-term cooperation. Several limitations should be noted. TF-IDF indicators are proxy measures of policy orientation rather than direct measures of preferences, and common method bias may exist because all variables are derived from the same textual source. In addition, cross-sectional data limit causal inference. Future research should employ longitudinal data, alternative indicators, or multilevel models to validate and extend these findings.
